# Acquired resistance to combined BET and CDK4/6 inhibition in triple-negative breast cancer

**DOI:** 10.1038/s41467-020-16170-3

**Published:** 2020-05-11

**Authors:** Jennifer Y. Ge, Shaokun Shu, Mijung Kwon, Bojana Jovanović, Katherine Murphy, Anushree Gulvady, Anne Fassl, Anne Trinh, Yanan Kuang, Grace A. Heavey, Adrienne Luoma, Cloud Paweletz, Aaron R. Thorner, Kai W. Wucherpfennig, Jun Qi, Myles Brown, Piotr Sicinski, Thomas O. McDonald, David Pellman, Franziska Michor, Kornelia Polyak

**Affiliations:** 1 Department of Medical Oncology, Dana-Farber Cancer Institute, Boston, MA 02215 USA; 2 Department of Data Sciences, Dana-Farber Cancer Institute, Boston, MA 02215 USA; 3 Harvard-MIT Division of Health Sciences and Technology, Harvard Medical School, Boston, MA 02115 USA; 4 Department of Medicine, Harvard Medical School, Boston, MA 02115 USA; 5 Department of Pediatric Oncology, Dana-Farber Cancer Institute, Boston, MA 02215 USA; 6 Department of Cell Biology, Harvard Medical School, Boston, MA 02115 USA; 7 Eli and Edythe L. Broad Institute, Cambridge, MA 02142 USA; 8 Department of Cancer Biology, Dana-Farber Cancer Institute, Boston, MA 02215 USA; 9 Department of Genetics, Harvard Medical School, Boston, MA 02115 USA; 10 Belfer Center for Applied Cancer Science, Dana-Farber Cancer Institute, Boston, MA 02215 USA; 11 Department of Cancer Immunology and Virology, Dana-Farber Cancer Institute, Boston, MA 02215 USA; 12 Department of Microbiology and Immunobiology, Harvard Medical School, Boston, MA 02115 USA; 13 Center for Cancer Genome Discovery, Dana-Farber Cancer Institute, Boston, MA 02215 USA; 14 Ludwig Center at Harvard, Harvard Medical School, Boston, MA 02115 USA; 15 Center for Functional Cancer Epigenetics, Dana-Farber Cancer Institute, Boston, MA 02215 USA; 16 Center for Cancer Evolution, Dana-Farber Cancer Institute, Boston, MA 02215 USA; 17 Department of Biostatistics, Harvard T. H. Chan School of Public Health, Boston, MA 02115 USA; 18 Department of Stem Cell and Regenerative Biology, Harvard University, Cambridge, MA 02138 USA; 19 Department of Pediatrics, Harvard Medical School, Boston, MA 02115 USA; 20 Present Address: Peking University Cancer Hospital and Institute, Beijing, 100142 China; 21 Present Address: Department of Life Science and the Research Center for Cellular Homeostasis, Ewha Womans University, Seoul, 120-750 Korea

**Keywords:** Cancer, Breast cancer, Cancer therapeutic resistance

## Abstract

BET inhibitors are promising therapeutic agents for the treatment of triple-negative breast cancer (TNBC), but the rapid emergence of resistance necessitates investigation of combination therapies and their effects on tumor evolution. Here, we show that palbociclib, a CDK4/6 inhibitor, and paclitaxel, a microtubule inhibitor, synergize with the BET inhibitor JQ1 in TNBC lines. High-complexity DNA barcoding and mathematical modeling indicate a high rate of de novo acquired resistance to these drugs relative to pre-existing resistance. We demonstrate that the combination of JQ1 and palbociclib induces cell division errors, which can increase the chance of developing aneuploidy. Characterizing acquired resistance to combination treatment at a single cell level shows heterogeneous mechanisms including activation of G1-S and senescence pathways. Our results establish a rationale for further investigation of combined BET and CDK4/6 inhibition in TNBC and suggest novel mechanisms of action for these drugs and new vulnerabilities in cells after emergence of resistance.

## Abstract (web summary)

Effective combination therapies to improve the efficacy of BET inhibitors are currently under investigation. Here, the authors examine palbociclib and paclitaxel as two promising candidates for combination therapies with BET inhibition in breast cancer and investigate the dynamics of resistance to these combinations through DNA barcoding and mathematical modelling.

## Introduction

Bromodomain and extra-terminal domain (BET) family proteins (BRD2, BRD3, BRD4, and BRDT) are epigenetic readers that regulate transcription, cell cycle, and cellular differentiation^1^. Specifically, BET proteins recognize acetylated lysines on histone tails and transcription factors, which are associated with open chromatin and transcriptional activation, and recruit various regulatory complexes, including other transcription factors, transcriptional coactivators, and chromatin modifiers^1,2^. In several cancer types, including multiple myeloma, leukemia, and lymphoma, BRD4 has been shown to drive transcription of key oncogenes such as *MYC* and *BCL2* by localizing to super-enhancers^2–5^. In the rare cancer NUT midline carcinoma, *BRD4* is even mutated itself to form a proto-oncogene^6^. Hence, BET proteins are critical to the function of oncogenic drivers in a variety of cancers. Recently, several small molecule inhibitors have been developed, including the prototypical JQ1, iBET151, and OTX015, that block the binding of BET proteins to acetylated histones, thereby inhibiting the expression of these oncogenes and subsequently cell proliferation^7–10^. BET inhibitors have thus received much interest as a new strategy to selectively target oncogenes that have otherwise been regarded as undruggable.

Previously, we and others have demonstrated the efficacy of BET inhibitors in triple-negative breast cancer (TNBC), an aggressive subtype of breast cancer that lacks targeted therapies^11,12^. However, cells can rapidly develop resistance to these drugs via multiple mechanisms, including bromodomain-independent chromatin binding of BRD4 through MED1 in TNBC^11^ and transcriptional activation via β-catenin in acute myeloid leukemia^13,14^. Therefore, effective combination therapies must be explored that can extend the efficacy of BET inhibitors and prevent or delay resistance.

A major obstacle to successfully treating cancer is the high degree of intratumor heterogeneity^15,16^, which can fuel tumor evolution and disease progression through selection for resistant subclones^17,18^. However, few studies have investigated the effects of treatment on tumor diversity and whether resistance is derived from subclones that existed prior to treatment or emerged during the course of therapy. It is critical to understand how the selective pressures of various therapies act on tumor cell populations, in order to better understand treatment outcome and manage progressive disease. Specifically, tumor evolution in the context of BET inhibition has never been studied.

Based on our previous work utilizing genetic screens, we identified two promising candidates for combination therapies with BET inhibition: palbociclib, a CDK4/6 inhibitor, and paclitaxel, a microtubule-inhibiting chemotherapy^19^. Here, we use high-complexity DNA barcoding and mathematical modeling to investigate the population dynamics of resistance to these drugs in combination with JQ1. Finally, we present genomic analyses to explore the mechanisms of cellular response and resistance.

## Results

### Palbociclib and paclitaxel synergize with JQ1

**Fig. 1:**
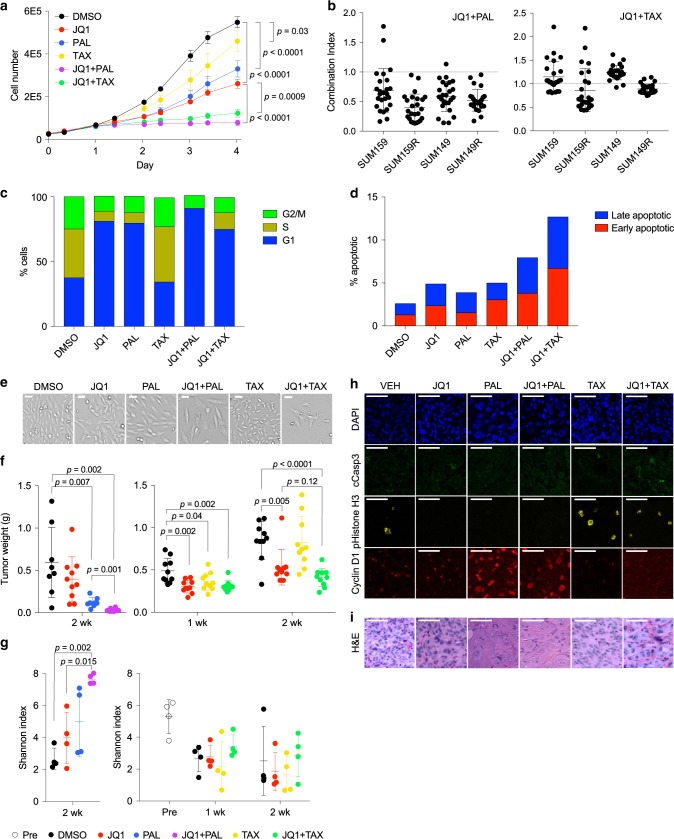
Palbociclib and paclitaxel synergize with JQ1 to induce cell-cycle arrest. **a** Growth curves of SUM159 cells treated in vitro with JQ1, palbociclib (PAL), and paclitaxel (TAX), alone and in combinations. Data are represented as mean ± SD, *n* = 3, one-way ANOVA followed by Sidak’s multiple comparisons test. Source data are provided as a Source Data file. **b** Levels of synergism between JQ1 and palbociclib (left) and paclitaxel (right) at various doses in parental lines (SUM159 and SUM149) and derived JQ1-resistant lines (SUM159R and SUM149R). Each point represents the combination index (CI) for one pair of concentrations, averaged over eight replicates. Mean ± SD are shown, CI = 1 additive, CI < 1 synergistic, and CI > 1 antagonistic. Source data are provided as a Source Data file. **c** Proportion of SUM159 cells in each cell-cycle phase following 24 h of treatment, determined by the Watson cell cycle model using flow cytometry on PI-stained SUM159 cells. *n* = 2. **d** Proportion of early apoptotic (annexin V^+^/PI^−^) and late apoptotic (annexin V^+^/PI^+^) SUM159 cells using flow cytometry following 3 days of treatment. *n* = 1. **e** Brightfield images of treated SUM159 cells. Images are representative of three independent experiments performed in triplicates. Scale bars represent 100 µm. **f** Tumor weights of SUM159 xenografts following 1 or 2 weeks of treatment. Mean ± SD are shown, *n* = 10, two-tailed Student’s *t*-test. Source data are provided as a Source Data file. **g** Shannon indices of barcode diversity of SUM159 xenografts before treatment and following 1 or 2 weeks of treatment. Mean ± SD are shown, *n* = 4, one-way ANOVA followed by Sidak’s multiple comparisons test. **h** Immunofluorescence staining for cleaved caspase 3, pHistone H3, and cyclin D1 in xenografts following 2 weeks of treatment. Images are representative of one experiment performed with five mice per group each with bilateral tumors. Scale bars represent 50 µm. **i** Hematoxylin and eosin staining of SUM159 xenografts. Scale bars represent 50 µm.

To begin to characterize the response of TNBC cells, we first tested JQ1, palbociclib, and paclitaxel, alone and in combinations in vitro. We found that both JQ1 + palbociclib and JQ1 + paclitaxel inhibited growth of SUM159 cells significantly more than any of the three drugs alone (Fig. 1a). We next tested each combination over a range of concentrations to determine whether the drug interactions were additive, synergistic, or antagonistic. JQ1 + palbociclib was strongly synergistic in two TNBC lines, SUM159 and SUM149, and even more so in their JQ1-resistant derivatives, SUM159R and SUM149R (Fig. 1b). On the other hand, JQ1 + paclitaxel was additive or antagonistic in the parental lines but likewise was more synergistic in the JQ1-resistant lines (Fig. 1b). Flow-cytometry analysis of cell cycle revealed that both JQ1 and palbociclib arrested cells in G1 phase, with a higher G1 fraction following treatment with both drugs combined than with either alone (Fig. 1c and Supplementary Fig. 1a, b). Apoptosis levels were also increased in both combination treatments, particularly with JQ1 + paclitaxel, while each single treatment only had a minimal effect (Fig. 1d and Supplementary Fig. 1c). In addition, cell morphology was noticeably altered, with cells becoming enlarged following treatment with JQ1 and palbociclib, especially the combination, as compared with DMSO treatment; there were also more apoptotic cells following treatment with JQ1 + paclitaxel (Fig. 1e). Thus, both palbociclib and paclitaxel combined with JQ1 induce significant cell-cycle arrest with moderate increases in apoptosis.

To investigate how intratumor heterogeneity is affected by these treatments, we labeled cells with the ClonTracer library^20^, where each cell is lentivirally infected with a unique DNA barcode, allowing us to follow the population’s clonality over time. We chose to focus our further studies on the SUM159 line due to its rapid growth rate and near-diploid genome with limited copy-number alterations, and its origin as an invasive ductal carcinoma, as compared with SUM149 which was derived from a *BRCA1*-mutant inflammatory breast cancer. Barcoded SUM159 cells were injected orthotopically into immunodeficient NOG mice, which were then treated for up to two weeks with JQ1, palbociclib, and paclitaxel, alone or in combinations. We found that JQ1 + palbociclib halted tumor growth, with significantly smaller tumor sizes than either of the single agents (Fig. 1f), thus confirming the efficacy of JQ1 + palbociclib in vivo. JQ1 + paclitaxel tended to inhibit tumor growth more than either drug alone, although the difference was not statistically significant (Fig. 1f).

We then performed barcode sequencing on the pre- and post-treatment tumors. We found that tumor diversity as measured by the Shannon index was higher in tumors treated with the combination therapies compared to those treated with either drug alone, which in turn was higher than those treated with vehicle (Fig. 1g). Indeed, tumors that were untreated or treated with single agents had a shift in their barcode frequency distributions, indicating that there were fewer barcodes making up a larger proportion of the population (Supplementary Fig. 2a). This observation suggests that these drugs had a primarily cytostatic effect in vivo, where the combination treatments inhibited the growth of all cells in the population and thus maintained the initial tumor diversity. Indeed, immunofluorescence staining for cyclin D1, phospho-histone H3, and cleaved caspase 3 showed that palbociclib and paclitaxel arrested cells in G1 and M phase, respectively, without inducing a significant amount of apoptosis (Fig. 1h). Moreover, the Shannon index was negatively correlated with tumor weights in untreated animals, which is consistent with selection for the fittest clones (Supplementary Fig. 2b). In addition, we again observed that tumor cells had strikingly altered morphology following treatment. In response to palbociclib, JQ1 + palbociclib, and to some extent JQ1 + paclitaxel, cells became enlarged, with decreased nuclear-cytoplasmic ratio and multiple irregular hypochromatic nuclei (Fig. 1i). These changes might indicate cellular senescence with chromatin reorganization or perturbed cell division. Together, these results indicate that the antitumor effects of JQ1 + paclitaxel and JQ1 + palbociclib are primarily from inhibition of growth, with modest effects on apoptosis.

### Barcode selection with long-term JQ1 combination treatments

Next, we asked how the barcodes selected by the treatments compared across multiple replicates. However, we did not observe resistance in vivo within a 2-week treatment period. In addition, we were not able to compare the shared barcodes between xenografts because the tumors that developed had mostly unique barcodes (Supplementary Fig. 2c), indicating that there was already selection for different clones that would graft in individual mice prior to treatment and thus they were not comparable. Therefore, we passaged the barcoded SUM159 cells in vitro with JQ1, palbociclib, paclitaxel, JQ1 + paclitaxel, or JQ1 + palbociclib for up to 18 passages. This approach allowed us to examine how clonality changes over a longer-term treatment with the development of resistance.

**Fig. 2:**
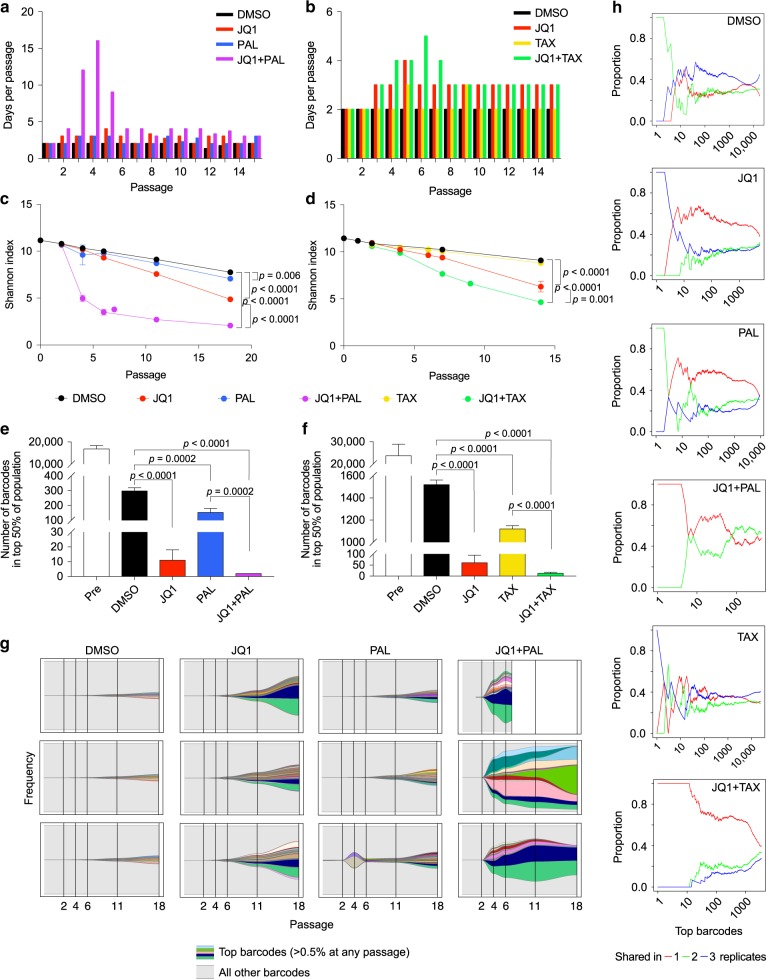
Long-term treatment with JQ1 combinations selects for both clones with pre-existing and acquired resistance. **a** Number of days between passages of barcoded SUM159 cells in DMSO, JQ1, palbociclib (PAL), and JQ1 + palbociclib. *n* = 3, except JQ1 + palbociclib in which *n* = 2 after passage 7. **b** Number of days between passages of barcoded SUM159 cells in DMSO, JQ1, paclitaxel (TAX), and JQ1 + paclitaxel. *n* = 3. **c** Shannon indices of diversity of barcodes in SUM159 cells passaged in JQ1 and palbociclib alone and in combination. Data are represented as mean ± SD, *n* = 3 except JQ1 + palbociclib in which *n* = 2 after passage 7, one-way ANOVA followed by Sidak’s multiple comparisons test. **d** Shannon indices of diversity of barcodes in SUM159 cells passaged in JQ1 and paclitaxel alone and in combination. Data are represented as mean ± SD, *n* = 3, one-way ANOVA followed by Sidak’s multiple comparisons test. **e** Number of barcodes making up the top 50% of the cell population before treatment and at the last passage of JQ1 and palbociclib. Data are represented as mean ± SD, *n* = 3 except JQ1 + palbociclib in which *n* = 2, one-way ANOVA followed by Sidak’s multiple comparisons test. **f** Number of barcodes making up the top 50% of the cell population before treatment and at the last passage of JQ1 and paclitaxel. Data are represented as mean ± SD, *n* = 3, one-way ANOVA followed by Sidak’s multiple comparisons test. **g** Frequencies of top barcodes (those representing at least 0.5% of the population at any passage) in cell populations selected with JQ1 and palbociclib, alone and in combination. Colors represent unique barcodes, gray background represents all other barcodes in the population, and each plot represents one replicate. Frequencies of all barcodes (*y*-axis) add up to 100%. **h** Representative plots of proportions of top barcodes ranked by frequency (*x*-axis) in one replicate at the last passage that are unique to that replicate, shared between two replicates, or shared between all three replicates in each treatment group.

We observed that the growth rate of treated cells initially slowed, particularly in combination-treated groups, but then increased again after several passages, suggesting a population bottleneck due to treatment selection (Fig. 2a, b). Interestingly, one replicate treated with JQ1 + palbociclib died out at passage 9. Barcode sequencing revealed that the diversity decreased in all groups over time but more in single treatments compared to DMSO and even more in combination-treated samples (Fig. 2c, d). Cell populations treated with JQ1 + palbociclib and JQ1 + paclitaxel also had the fastest shifts in their barcode distributions (Supplementary Fig. 2d, e). By the last passage, only 2 and 13 barcodes on average made up the top 50% of the populations, respectively (Fig. 2e, f). In particular, the Shannon index for JQ1 + palbociclib-treated samples had the sharpest drop, indicating that this treatment generated the strongest selective pressure (Fig. 2c).

We then compared the barcodes that were selected between replicates to see whether resistance was likely to be pre-existing or acquired de novo. We expected that clones with pre-existing resistance would be shared among multiple replicates, whereas those that acquired resistance during treatment would be unique to individual replicates. We found that following selection in JQ1 or palbociclib, most of the barcodes were unique to individual replicates, although a few were shared amongst the top barcodes (Fig. 2g, h and Supplementary Fig. 3a), suggesting that treatment selects for both clones with pre-existing and acquired resistance. However, in the JQ1 + palbociclib- and JQ1 + paclitaxel-selected populations, there were more barcodes that were unique rather than shared, particularly among the top barcodes (Fig. 2g, h and Supplementary Fig. 2f and 3a, b). The fact that the same barcodes were not selected for between replicates suggests that resistance to the combination treatments is rare in the initial population and is more likely to be acquired. Interestingly, the JQ1 + palbociclib replicate that died out had a very similar barcode composition to one of the replicates that continued to proliferate (Fig. 2g), which we hypothesized was due to an acquired phenotype.

### Mathematical modeling is consistent with acquired resistance

**Fig. 3:**
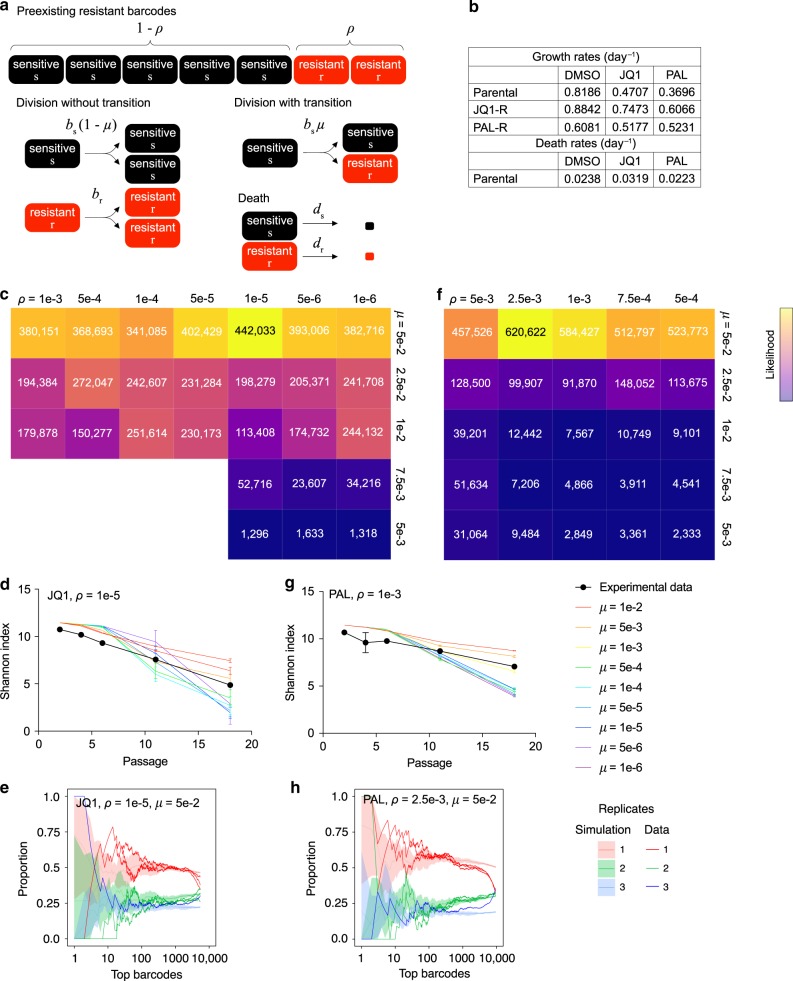
Mathematical modeling of resistance to JQ1 and palbociclib. **a** Design of the two-type birth-death process to model barcode evolution during treatment with JQ1 or palbociclib. See Methods for model details. **b** Experimentally determined growth and death rates used in the model. Source data are provided as a Source Data file. **c** Heatmap of likelihood scores for each pair of parameters tested to simulate cell populations passaged in JQ1. **d** Comparison between simulated data and experimental data of Shannon indices for cells passaged in JQ1, for the best-fit value of *ρ* and various values of *μ*. Data are represented as mean ± SD, *n* = 3. **e** Comparison between simulated data and experimental data of proportions of top barcodes (*x*-axis) that are unique, shared between two replicates, or shared between all three replicates at the last passage, with the best-fit parameters for JQ1. Simulated distributions are represented as mean ± SD, *n* = 5 independent simulations with three replicates each. **f** Heatmap of likelihood scores for each pair of parameters tested to simulate cell populations passaged in palbociclib. **g** Comparison between simulated data and experimental data of Shannon indices for cells passaged in palbociclib, for the best-fit value of *ρ* and various values of *μ*. Data are represented as mean ± SD, *n* = 3. **h** Comparison between simulated data and experimental data of proportions of top barcodes (*x*-axis) that are unique, shared between two replicates, or shared between all three replicates at the last passage, with the best-fit parameters for palbociclib. Simulated distributions are represented as mean ± SD, *n* = 5 independent simulations with three replicates each.

To computationally infer the extent of pre-existing vs. acquired resistance, we designed a mathematical model that simulates barcode selection in order to test various rates of resistance. We used a birth-death process model comprising sensitive and resistant cells, with an initial proportion of barcodes *ρ* being resistant prior to therapy (Fig. 3a). Sensitive and resistant cells have individual birth (*b*_s_ and *b*_r_) and death rates (*d*_s_ and *d*_r_), and at each division, a sensitive cell can acquire resistance by giving rise to a resistant daughter cell at a transition rate *μ*. The growth rates for each cell type were experimentally measured using resistant lines derived from the post-selection pools, while death rates were estimated from previous flow cytometry data (Fig. 3b and Fig. 1d). We used the barcode distributions of the pre-treatment samples to estimate the initial barcode frequencies. Replicate plates were sampled from this initial pool, and proliferation was simulated for 18 passages, as in the in vitro experiments, for a range of parameters for *ρ* and *μ*. We evaluated the simulation results by comparing them with the experimental data using their Shannon indices of diversity and proportions of shared barcodes between replicates (see Methods for details).

We performed simulations with *ρ* ranging from 1 × 10^−1^ to 1 × 10^−6^ and *μ* ranging from 1 × 10^−2^ to 1 × 10^−6^, focusing on JQ1 and palbociclib. We found that several parameter combinations fit the Shannon indices of the experimental data, including high rates of *ρ* and/or high rates of *μ* (Supplementary Fig. 3c, d). However, a comparison of the proportion of shared barcodes in the simulations vs. data narrowed down the parameter search space (Supplementary Fig. 4a, b). Notably, only a high transition rate could match the proportion of unique barcodes observed experimentally. Using a likelihood score that compared the distributions over five independent simulation runs to the experimental data, we found that the best-fit parameters for JQ1 were *ρ* = 1 × 10^−5^ and *μ* = 5 × 10^−2^ (Fig. 3c–e and Supplementary Fig. 5a), while the best fit for palbociclib was *ρ* = 2.5 × 10^−3^ and *μ* = 5 × 10^−2^ (Fig. 3f–h and Supplementary Fig. 5b). In other words, 1 in 100,000 and 1 in 400 cells have pre-existing resistance to JQ1 and palbociclib, respectively, while a cell acquires resistance to either drug in 1 in 20 divisions. Although 5 × 10^−2^ was the highest value tested for *μ* and the best-fit rate might be higher, these results are still consistent with a higher level of acquired resistance to both JQ1 and palbociclib relative to pre-existing resistance.

### Rb loss is one mechanism of resistance to JQ1 + palbociclib

**Fig. 4:**
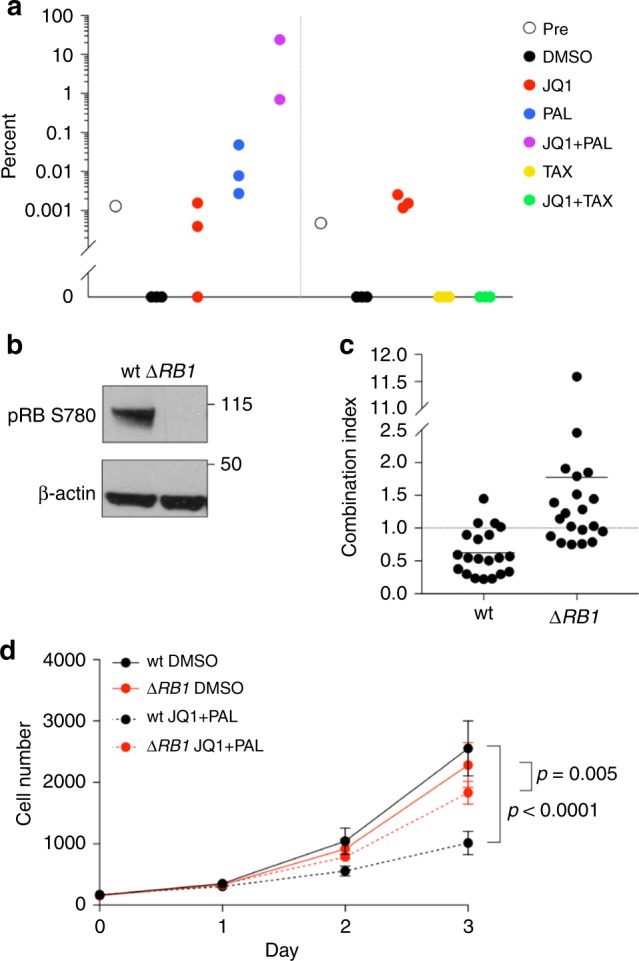
Rb loss is one possible mechanism of resistance to JQ1 + palbociclib. **a** Frequency of *RB1* E864* mutation detected by ddPCR in pre-treatment cell populations and after selection with JQ1, palbociclib, and paclitaxel, alone and in combinations. Source data are provided as a Source Data file. **b** Western blots show pRB levels in wild-type and *RB1*-deleted SUM159 cells. **c** Levels of synergism between JQ1 and palbociclib in wild-type and *RB1*-deleted cells at various doses. Each point represents the combination index (CI) for one pair of concentrations, averaged over eight replicates. CI = 1 additive, CI < 1 synergistic, and CI > 1 antagonistic. Source data are provided as a Source Data file. **d** Growth curves of wild-type and *RB1*-deleted cells treated with DMSO or 77 nM JQ1 + 611 nM palbociclib. Data are represented as mean ± SD, *n* = 68 for DMSO and *n* = 8 for JQ1 + palbociclib, one-way ANOVA followed by Sidak’s multiple comparisons test. Source data are provided as a Source Data file.

Next, we investigated mechanisms of resistance by performing exome sequencing on pre- and post-selection cells. Loss of function of Rb, a key inhibitor of G1-S progression, is known to cause resistance to palbociclib in ER-positive breast cancer^21,22^. Consistent with these previous findings, we detected a nonsense mutation in *RB1* (E864*) in JQ1 + palbociclib-selected cells, at a frequency of 27%, which was not detected in any of the other cell populations (Supplementary Data 1). We then used droplet digital PCR (ddPCR) to look for the mutation in rare pre-existing clones in the pre-treatment population and in rare cells in the other post-selection groups. We found that indeed this *RB1* mutation was present in the pre-treatment pool, at a frequency of 1 in 100,000 (Fig. 4a). We also found the mutation in some replicates selected with JQ1 or palbociclib alone, but it either remained at the same frequency or expanded to at most 0.05% in one palbociclib replicate (Fig. 4a). Interestingly, the E864* mutation appeared at different frequencies in the two JQ1 + palbociclib replicates (24.1% and 0.7%, Fig. 4a). Thus, we concluded that Rb loss is not necessary for resistance to JQ1 + palbociclib but represents one possible mechanism.

To validate that Rb is functionally relevant, we deleted *RB1* in SUM159 cells using CRISPR (Fig. 4b) and treated them with the drug combination. We found that *RB1*-deleted cells gained resistance to JQ1 + palbociclib and that the two drugs were no longer synergistic but merely additive (Fig. 4c, d). Thus, loss of Rb can confer resistance to the JQ1 + palbociclib combination and increases cellular fitness during treatment.

### JQ1 and palbociclib induce increased ploidy

**Fig. 5:**
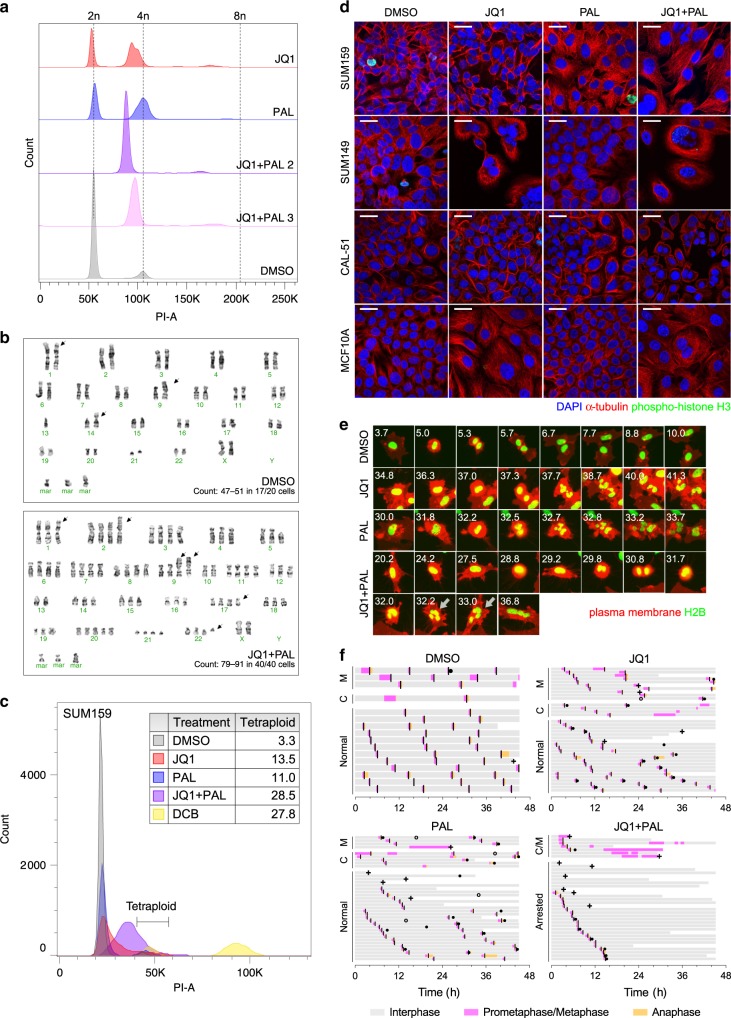
JQ1 and palbociclib induce increased ploidy through cell division failure. **a** Histograms of DNA content by flow cytometry in PI-stained post-selection SUM159 cells. **b** Representative karyotypes of post-selection SUM159 cells after passaging in DMSO (top) and in JQ1 + palbociclib (bottom). Ranges of chromosome numbers and numbers of metaphase spreads counted are shown. Arrows indicate chromosomal abnormalities. **c** Histogram of DNA content by flow cytometry in Hoechst-stained FUCCI-labeled G1 SUM159 cells following 7 days of treatment. Dihydrocytochalasin B (DCB)-induced tetraploid cells were used as a positive control. Table indicates proportions of cells in the tetraploid gate. **d** Immunofluorescence staining of α-tubulin and phospho-histone H3 with DAPI in SUM159, SUM149, CAL-51, and MCF10A cells following 7 days of treatment. Images are representative of three independent experiments. Scale bars represent 25 µm. **e** Time-lapse live cell imaging of SUM159 cells with fluorescently labeled H2B and plasma membrane undergoing mitosis during treatment with DMSO, JQ1, palbociclib, and JQ1 + palbociclib. Numbers indicate hours following start of treatment. Arrows indicate failed cytokinesis. Images are representative of three independent experiments. **f** Cell cycle phase lengths for individual cells during time-lapse imaging period. Each bar represents one single cell. Line indicates onset of cytokinesis. Chromosomal missegregation (closed circle), apoptosis (plus sign), and fusion (open circle) events are marked. Time indicates hours following start of treatment. M mitotic delay (prometaphase/metaphase > 1 h), C cytokinesis failure, A arrested.

We then investigated whether our post-selection cells had any alterations in genome copy number. Flow cytometry analysis revealed that JQ1, palbociclib, and JQ1 + palbociclib-selected cells had an increased fraction that was approximately 4n, as well as gain of a small 8n peak (Fig. 5a). This change was most significant in JQ1 + palbociclib, which had no cells at 2n (Fig. 5a). Interestingly the peaks in the resistant populations were centered at slightly less than 4n and were broader than the peaks in the DMSO-selected samples (Fig. 5a), suggesting that these genomes had likely arisen through tetraploidization followed by chromosomal losses, leading to heterogeneity in chromosomal copy numbers. We confirmed the ploidy findings with karyotyping, which showed that JQ1, palbociclib, and JQ1 + palbociclib-selected cells had four copies of nearly all chromosomes, with some chromosomes undergoing further losses or gains to 2–6 copies (Fig. 5b and Supplementary Fig. 6a). On the other hand, cells passaged in DMSO were primarily near-diploid (Fig. 5b), although three out of 20 cells counted were also found to be tetraploid (Supplementary Fig. 6a).

We thus asked whether this tetraploidy was induced by the treatments or by clonal expansion of pre-existing tetraploid clones. In order to distinguish tetraploids cells from diploids in G2/M, we labeled SUM159 cells with a fluorescence ubiquitination cell cycle indicator (FUCCI) and used flow cytometry to assess only the G1 fraction (Supplementary Fig. 6b). We found that within 7 days, almost all cells treated with JQ1 + palbociclib had a DNA content of more than 2n with nearly one-third being tetraploid (Fig. 5c). We also observed a significant increase in DNA content after JQ1 and palbociclib treatment in other TNBC cell lines, SUM149 and CAL-51, and in non-tumorigenic immortalized breast epithelial cells MCF10A labeled with the FUCCI reporter (Supplementary Fig. 6c). Of note, while there were smaller percentages of SUM149 and CAL-51 cells within the tetraploid gate, there were actually more cells that exceeded 4n. We further tested a panel of 9 unlabeled cell lines of different TNBC subtypes (i.e., luminal, basal, and mesenchymal) and ploidy and observed an increase in DNA content in a majority of them following JQ1 and palbociclib treatment; cell lines that were already hyperdiploid appeared to acquire even higher levels of ploidy (Supplementary Fig. 6d, e). This increase in chromosome numbers was confirmed in metaphase spreads of treated SUM159 cells (Supplementary Fig. 7a). Likewise, there was an increase in ploidy in cells treated with other CDK4/6 inhibitors, abemaciclib and ribociclib, combined with JQ1 (Supplementary Fig. 7b). However, ploidy was not affected by siRNA knockdown of *CDK4*, *CDK6*, or both (Supplementary Fig. 7c, d). Therefore, the induction of polyploidy was not specific to SUM159 cells or to palbociclib but may depend on inhibition of the whole cyclin D-CDK4/6 complex.

We ruled out cell fusion as a major mechanism of this increased ploidy by co-culturing GFP- and RFP-labeled SUM159 cells and looking for yellow (GFP^+^RFP^+^) cells, which could only have arisen through fusions of red and green cells (Supplementary Fig. 7e–g). We did find double-positive cells at a low rate (<1%), but the fraction remained relatively unchanged after 7 days of treatment (Supplementary Fig. 7g).

To investigate whether polyploid cells are inherently more drug-resistant, we used fluorescence-activated cell sorting (FACS) to enrich for spontaneously occurring tetraploids by sorting for SUM159-FUCCI G1 4n cells and GFP^+^RFP^+^ cells. However, they did not have any differences in sensitivity to JQ1 or palbociclib compared with parental FUCCI-labeled or unlabeled lines (Supplementary Fig. 7h), nor did they have any decreased synergy over a range of concentrations (Supplementary Fig. 7i). Furthermore, we previously generated and described homofusions of SUM159 cells that are tetraploid^23^. When these cells were treated with JQ1 and palbociclib, octaploid cells were produced (Supplementary Fig. 7j). Therefore, we concluded that genome doubling is induced directly by JQ1 and palbociclib and hypothesized that it was arising through cell division failure. Indeed, many of the cells became multinucleated following combination treatment, suggesting that they complete mitosis without cytokinesis (Fig. 5d).

To further investigate the mechanism of this whole-genome doubling, we performed time-lapse live cell imaging for 2 days on treated SUM159 cells with fluorescently labeled histone H2B and plasma membrane. We observed that, in addition to a decreased division rate, there were a variety of errors in cell division, which began within a few hours of adding the drugs. Compared with untreated cells that mostly divided normally (Fig. 5e and Supplementary Video 1), JQ1 and palbociclib both caused chromosomal segregation errors, where cells further divided their chromosomes following anaphase, forming bi- or multinucleated cells (Fig. 5e and Supplementary Videos 2–3). More cells also exhibited mitotic delays, which were often coupled with failure to complete mitosis, absence of cytokinesis, and appearance of micronuclei (Fig. 5f and Supplementary Video 4). This was most significant in the JQ1 + palbociclib combination, where most of the cells that experienced mitotic delay did not initiate anaphase even after many hours in prometaphase/metaphase and eventually reverted to interphase without karyokinesis (Fig. 5e, f and Supplementary Videos 5–6). Furthermore, there was a modest increase in cell death and infrequent cell fusion events, consistent with our other data (Fig. 5f). Thus, we concluded that JQ1 and palbociclib can induce increased ploidy and aneuploidy through chromosomal missegregation or cytokinesis failure, but their combination disrupts cells even earlier in the cell cycle, blocking them from initiating anaphase. While we observed these ploidy changes in multiple TNBC cell lines, the physiologic relevance of this finding requires further analyses in TNBC patients.

### G1-S genes are upregulated in JQ1 + palbociclib resistance

**Fig. 6:**
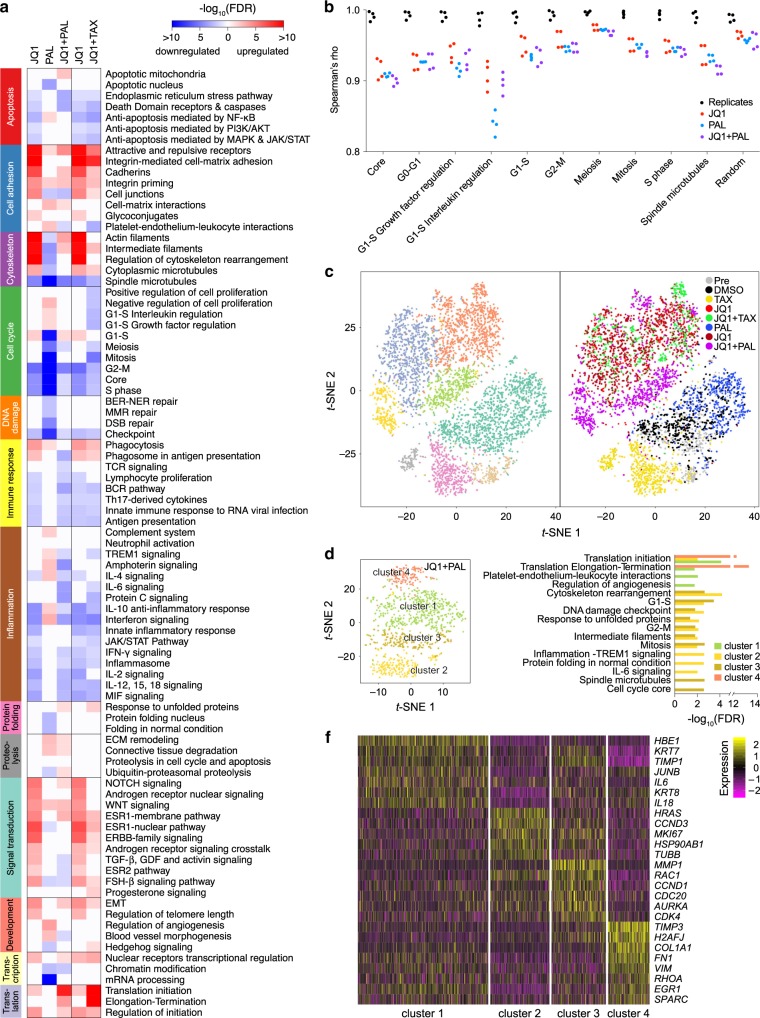
G1-S pathways are upregulated in JQ1 + palbociclib-resistant cells. **a** Enriched process networks in differentially expressed genes by bulk RNA-seq of post-JQ1, palbociclib, JQ1 + palbociclib, and JQ1 + paclitaxel-selected cells compared with post-DMSO-selected cells. **b** Spearman’s rank correlation coefficient (rho) for bulk expression of genes in cell cycle related process networks. Black indicates comparison between replicates in post-DMSO, JQ1, palbociclib, and JQ1 + palbociclib-selected groups. Red, blue, and purple indicate all pairwise comparisons between post JQ1, palbociclib, and JQ1 + palbociclib-selected cells with post-DMSO-selected cells. **c** *t*-SNE plots of cells from the pre-selection and each post-selection population by single-cell RNA-seq, colored by cluster (left) and by treatment (right). Each point represents one single cell. **d** *t*-SNE plot of cells from only the JQ1 + palbociclib-selected population by single-cell RNA-seq colored by cluster. Each point represents one single cell. **e** Enriched process networks in differentially expressed genes in each cluster of the JQ1 + palbociclib-selected population by single-cell RNA-seq. **f** Heatmap of differentially expressed genes in each cluster in the JQ1 + palbociclib-selected population by single-cell RNA-seq. Each column represents one single cell.

Since BET proteins function as transcriptional regulators, we examined the gene expression using RNA-seq for changes associated with drug resistance in the post-selection cells. We found that G1-S pathways were upregulated in JQ1 and JQ1 + palbociclib-selected cells compared to DMSO-treated cells (Fig. 6a). In particular, *CCND1* and *CCNE* expression were increased in JQ1 + palbociclib compared with JQ1 and in JQ1 compared with DMSO, while *CDKN1A* (p21) and *RB1* were also decreased in the combination compared with JQ1 (Supplementary Fig. 8a and Supplementary Data 2). This observation suggests that genes involved in the G1-S transition are important for escaping JQ1 + palbociclib-induced growth arrest. In addition, *MYC* and *BCL2L1* were more highly expressed in the combination compared to JQ1 alone and to DMSO (Supplementary Fig. 8a and Supplementary Data 2), consistent with our previous work that identified them as gained super-enhancers in the derived JQ1-resistant line SUM159R^11^. Furthermore, a set of DNA replication (*ORC2*, *ORC5*, *MCM8*, *TOP1*, and *WRN*) and chromosomal segregation (*ANAPC13*, *ANAPC2*, and *ZWILCH*) genes was upregulated in combination-selected cells, which was not observed in cells selected by either agent alone (Supplementary Fig. 8a and Supplementary Data 2). These results suggest that escape from G1 arrest and DNA stabilization mediate the adaptation to tetraploidy induced by JQ1 + palbociclib.

To determine if some of the gene expression changes were due to increased ploidy, we also analyzed SUM159 somatic cell fusions that were tetraploid^23^ but not treated with drugs. We found limited overlap of the differentially expressed genes between the two populations (Supplementary Fig. 8a). Thus, the gene expression pattern of cells resistant to JQ1 + palbociclib is not simply caused by increased ploidy.

Since BRD4 is known to be involved in the transcription of genes necessary for mitotic exit^24^, we hypothesized that JQ1 generates errors in mitosis by dysregulating the relative levels of cell cycle genes. Thus, we compared the rank correlations of gene expression with cell cycle-related gene lists, between and within treatment groups. We found that there was a decrease in Spearman’s rho for G0-G1, G1-S, and spindle microtubule genes after treatment with JQ1, palbociclib, or both compared with DMSO, indicating that their relative levels of gene expression became disproportionate (Fig. 6b). This observation suggests that JQ1 and palbociclib have effects on multiple steps of the cell cycle, including chromosomal segregation, which could lead to chromosomal instability during cell division. Furthermore, we performed single-cell RNA-seq to examine whether there was heterogeneity in the response to any of the treatments. Using *t*-Distributed Stochastic Neighbor Embedding (*t*-SNE) analysis, we found that most cells clustered by treatment (Fig. 6c), but JQ1 + palbociclib-resistant cells formed four distinct clusters, whereas the other post-selection populations had less heterogeneity (Fig. 6d and Supplementary Fig. 8b). Clusters 1 and 4 had increased activity of pathways related to protein translation and regulation of the microenvironment, including extracellular matrix and cytokine genes (e.g., *COL1A1*, *FN1*, *TIMP3*, *IL6*, and *IL18*) as well as histone variant H2A.J (*H2AFJ*), which is associated with cellular senescence (Fig. 6e, f and Supplementary Data 3). Thus, this gene expression pattern may be consistent with a senescence-associated secretory phenotype (SASP). Conversely, the other two clusters exhibited upregulation of cell cycle-related genes, including *CCND1* and *CDK4* in cluster 3 and *CCND3* in cluster 2 (Fig. 6e, f and Supplementary Data 3). In addition, the IL-6 signaling pathway was enriched in cluster 2, which may be in response to secreted IL-6 by cluster 1 (Fig. 6e, f and Supplementary Data 3). These distinct phenotypes indicate that cells can respond differently to the JQ1 + palbociclib combination and that there may be multiple mechanisms of resistance or clonal cooperation driving resistance.

### Drug schedule affects treatment outcome

**Fig. 7:**
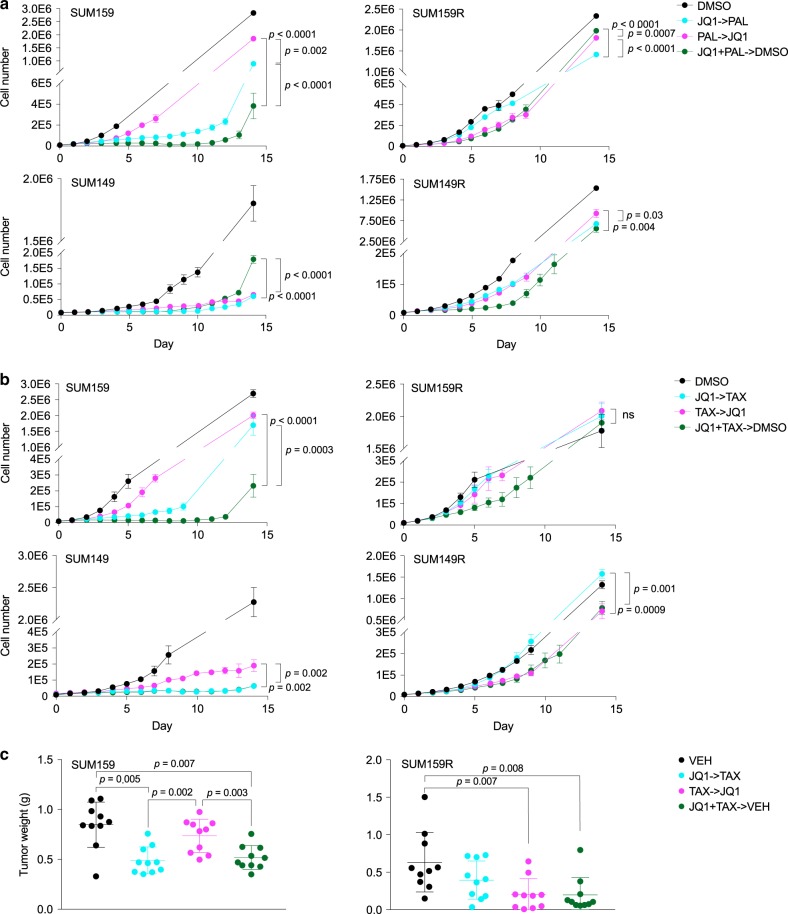
Drug schedule affects treatment outcome and varies between cell lines. **a** Growth curves of parental (SUM159 and SUM149) and derived JQ1-resistant (SUM159R and SUM149R) cell lines treated with JQ1 and palbociclib sequentially in either order or concomitantly followed by vehicle. Data are represented as mean ± SD, *n* = 3, one-way ANOVA followed by Tukey’s multiple comparisons test. **b** Growth curves of parental (SUM159 and SUM149) and derived JQ1-resistant (SUM159R and SUM149R) cell lines treated with JQ1 and paclitaxel sequentially in either order or concomitantly followed by vehicle. Data are represented as mean ± SD, *n* = 3, one-way ANOVA followed by Tukey’s multiple comparisons test. ns not significant. **c** Tumor weights of SUM159 and SUM159R xenografts following 2 weeks of treatment with JQ1 and paclitaxel sequentially in either order or concomitantly followed by vehicle. Mean ± SD are shown, *n* = 10, two-tailed Student’s *t*-test. Source data are provided as a Source Data file.

Lastly, we investigated whether the order of drug administration would affect treatment outcomes. Thus, we treated SUM159 and SUM149 parental cells and SUM159R and SUM149R JQ1-resistant derivatives sequentially with JQ1 for 1 week followed by palbociclib or paclitaxel for 1 week or the reverse order, or concurrently for 1 week followed by vehicle for 1 week. We found in all cell lines that JQ1 followed by palbociclib was superior to the reverse; however, upfront combination was best in SUM159 and SUM149R (Fig. 7a). With JQ1 and paclitaxel, we found that JQ1 followed by paclitaxel was superior in the parental cells, but the reverse was true in the resistant lines; however, in all cases, upfront combination of JQ1 + paclitaxel was equally or more effective than the better of the two sequential treatments (Fig. 7b). We observed the same result when treating NOG mice harboring SUM159 and SUM159R xenografts with JQ1 and paclitaxel (Fig. 7c). These data may reflect selection for or modulation of sensitivity to the second drug, as well as an increased cytotoxic effect of JQ1 + paclitaxel, where giving the most effective therapy upfront is best, compared with JQ1 + palbociclib, where prolonged inhibition of proliferation is beneficial.

## Discussion

In this study, we investigated the response of TNBC cells to BET combined with CDK4/6 inhibition using palbociclib and with microtubule inhibition using paclitaxel. We found that the combination treatments more effectively induced cell cycle arrest compared with single agents. Using DNA barcoding and mathematical modeling, we discovered that there is a high rate of acquired resistance to JQ1 and palbociclib relative to pre-existing resistance. Much to our surprise, we found that all of the JQ1 + palbociclib double resistant cells were near-tetraploid and that this drug combination is capable of inducing aneuploidy via errors in cell division. Using genomic and transcriptomic profiling, we found that resistant cells upregulated G1-S and senescence-associated genes, while downregulating genes involved in cell cycle inhibition, which may mediate the escape from growth arrest. One potential mechanism was selection for loss of Rb, but this was not found in a majority of the cells, which suggests that resistance is more likely to be acquired through alterations of gene expression.

To our knowledge, this is the first study examining the evolution of cell populations during treatment with JQ1 or palbociclib. The selection for *RB1*-mutant clones was due to a nonsense mutation, E864*, which has previously been reported in human tumors^25,26^. Rb loss is a well-known mechanism of resistance to palbociclib in ER-positive breast cancer^21,22^ and in our previous CRISPR knockout screens conferred resistance to JQ1^19^. We found that Rb loss could also confer resistance to the JQ1 + palbociclib combination, but the E864* mutation was not clonal, and thus loss of Rb is not necessary for resistance. We did not find exome mutations in other cell cycle genes, and the other mutations that arose are of unknown significance.

This is the first report of mathematical modeling of evolution of resistance to BET and CDK4/6 inhibitor treatments. The high rate of acquired resistance that we inferred compared with pre-existing resistance suggests epigenetic rather than genetic changes, which would be expected to be similar to the mutation rate of DNA replication. Indeed, we found that post-selection SUM159 cells had upregulation of G1-S pathways, including increased expression of cyclin E, which has previously been found to confer resistance to palbociclib^21^. However, the increased phenotypic heterogeneity we saw with single-cell RNA-seq suggests that there are multiple mechanisms of resistance to JQ1 + palbociclib, which is also consistent with an acquired mode of resistance, since each clone would have to develop resistance independently. The gene expression pattern seen in two of the clusters was consistent with SASP, while another cluster exhibited increased cytokine signaling response. Thus, clonal cooperation through these secreted factors may promote growth of the whole population. Indeed, minor subpopulations have been shown to be capable of driving tumor growth through such non-cell-autonomous mechanisms^27^. Polyclonal resistance could pose a challenge to treatment, but these drivers, potentially senescent cells, could be targeted to trigger tumor collapse.

We observed that ploidy increases upon treatment with JQ1 + palbociclib through errors in mitosis and cytokinesis. We also determined that both drugs alone could disrupt expression of mitosis genes and induce tetraploidy, albeit at a lower rate compared with their combination. Multinucleation has previously been observed with JQ1 through direct suppression of Aurora kinase^12^. While induction of tetraploidy by palbociclib has not been reported, activity of the cyclin D-CDK4/6 complex is known to be involved in programmed polyploidy in megakaryocytes and duplication of centrosomes^28,29^. Furthermore, chemotherapeutic treatment of colorectal cancer cells with homozygous deletion of *CDKN1A* encoding the p21 CDK inhibitor leads to increased ploidy^30^. Thus, we propose that aneuploidy-induced arrest resulting from cell division failure contributes to the anti-proliferative effects of BET and CDK4/6 inhibition.

Further investigation will be needed to elucidate this unexpected mechanism of action. Evidence suggests that tetraploidy is more prone to arise in p53-null cells and that subsequent arrest can be due to damage to the spindle, cytoskeleton, or DNA, dependent on p53^31–34^. However, we have also found JQ1 and palbociclib to increase ploidy in CAL-51 and MCF10A, which have wild-type *TP53*; thus, this effect is not exclusive to p53 loss. In patient tumor samples, tetraploidy has been associated with not only *TP53* mutations but also *CCNE1* amplification and *RB1* loss^35^, suggesting that it is propagated by defects in G1 arrest, consistent with our model. Besides its canonical role as a cell cycle suppressor, Rb is also thought to preserve genomic stability through regulation of spindle assembly, chromosome segregation, and DNA replication^36,37^. Similarly, regulation of cyclin E is required for proper centrosome duplication, and overexpression has been shown to result in accumulation of aneuploid cells^38,39^. Cyclin D1 and D2 have also been reported to mediate tolerance to genome doubling^40,41^. Therefore, the genomic and transcriptional changes found in our resistant cells may have led not only to increased proliferation but also to a state permissive to aneuploidy. The decision of cells to arrest, continue cycling, or undergo apoptosis following JQ1 + palbociclib-induced genomic alterations requires further study. Interestingly, *MYC*, a known target of BRD4, was upregulated in our post-selection cells but has been found to influence mitotic cell fate in the direction of death in mitosis over slippage^42^. The low rate of apoptosis in our cells may be due to upregulation of Bcl-xL (*BCL2L1*), tipping the scale in favor of survival. Additional work will be needed to understand how cells adapt to the increased copy number and avoid further genome doublings.

Whole-genome doubling is observed in one-third of human cancers, and tetraploidy has been associated with tumorigenesis and poor prognosis^34,35,43^. It has been proposed that tetraploidy leads to increased tolerance of DNA damage and chromosomal instability and thus accelerates the rate of tumor evolution^31,43^. Thus, aneuploidy induced by JQ1 + palbociclib may have important implications for tumor progression, which will need to be addressed in future studies. It is possible that the adaptations to increased ploidy required for resistance may result in increased heterogeneity, posing a challenge to subsequent clinical management. However, the increased cellular stress imposed by tetraploidy may sensitize those tumors to unique vulnerabilities, such as centrosome and genomic instability or proteotoxic and metabolic stress^44–47^. Whole-genome doubling has been directly linked to tumor initiation, resulting from genomic rearrangements and malignant transformation^32–34^ but has also been shown to have a tumor-suppressive role in hepatocytes^48^. Thus, its effects are likely to be cell type- and context-dependent, making tetraploidy a double-edged sword that could either trigger growth arrest or tumorigenesis. The consequences of tetraploidy in normal tissues induced by JQ1 + palbociclib as well as by other therapeutics require further study.

Our findings have direct translational impact and clinical significance. TNBC is an aggressive disease, associated with younger age and worse prognosis than other subtypes of breast cancer, and novel targeted therapies for this disease are still lacking. CDK4/6 inhibitors have been approved for advanced ER-positive breast cancer but are thought to be ineffective in basal-like disease^49^. Nevertheless, we have found that palbociclib greatly improved the sensitivity to JQ1 and thus may be useful in TNBC. The effect of the drugs’ induction of aneuploidy in promoting further tumorigenesis is unknown; however, the current median survival for metastatic TNBC is 13 months^50^, so the risk for an eventual secondary malignancy may not be relevant for these patients. Our study provides rationale for further preclinical and clinical investigation of this combination.

## Methods

### Cell lines

SUM159 and SUM149 breast cancer cells were obtained from Steve Ethier (University of Michigan) and cultured in 50% DMEM/F12, 45% Human Mammary Epithelial Cell Growth Medium, and 5% FBS, with 1% Pen Strep (Thermo Fisher Scientific). JQ1-resistant SUM159R and SUM149R lines were previously described^11^ and also cultured in the presence of 20 µM and 10 µM JQ1, respectively. SUM159 homofusions were previously described^23^. SUM229 and SUM185 cells were obtained from Steve Ethier (University of Michigan) and cultured in DMEM/F12 with 5% FBS, 1 µg/mL hydrocortisone, 5 µg/mL insulin, and 1% Pen Strep. CAL-51 cells were obtained from DSMZ and cultured in DMEM with 20% FBS and 1% Pen Strep. MCF10A cells were obtained from ATCC and cultured in DMEM/F12 with 5% horse serum, 10 µg/mL insulin, 20 ng/mL EGF, 0.5 µg/mL hydrocortisone, 0.1 µg/mL cholera toxin, and 1% Pen Strep. EMG3 cells were obtained from Eva Matouskova (Academy of Sciences of the Czech Republic) and cultured in DMEM/F12 with 10% FBS, 10 µg/mL insulin, 20 ng/mL EGF, 0.5 µg/mL hydrocortisone, 0.1 µg/mL cholera toxin, and 1% Pen Strep. Hs578T cells were obtained from ATCC and cultured in DMEM with 10% FBS, 10 µg/mL insulin, and 1% Pen Strep. MDA-MB-157 cells were obtained from ATCC and cultured in McCoy’s Media with 10% FBS and 1% Pen Strep. MDA-MB-436 cells were obtained from ATCC and cultured in McCoy’s Media with 10% FBS, 10 µg/mL insulin, and 1% Pen Strep. UACC3199 cells were obtained from University of Arizona, HCC1143 from ATCC, and HCC2185 from Adi Gazdar (UT Southwestern) and were cultured in RPMI with 10% FBS and 1% Pen Strep. The identity of the cell lines was confirmed based on STR and exome-seq analyses. Cells were regularly tested for mycoplasma.

### Animals

In vivo studies were conducted using 6-week-old female immunodeficient NOD.Cg-*Prkdc*^*scid*^*Il2rg*^*tm1Sug*^/JicTac (NOG) mice (Taconic) or NOD.Cg-*Prkdc*^*scid*^*Il2rg*^*tmWjl*^/SzJ (NSG) mice (Jackson Laboratory). Animal studies were performed according to protocol 11-023 or by the Lurie Family Imaging Center according to protocol 04-111, approved by the Dana-Farber Cancer Institute Animal Care and Use Committee.

### Inhibitor treatments

For proliferation, cell cycle, apoptosis, barcode selection, and ploidy experiments, SUM159 cells were treated with 100 nM JQ1, 160 nM palbociclib, and 0.6 nM paclitaxel. For flow cytometry studies of ploidy, all cells were treated with 250 nM JQ1 and 500 nM palbociclib for 7 days and 5 µM dihydrocytochalasin B (DCB) overnight. SUM159 cells were also treated with 500 nM ribociclib or abemaciclib.

### Proliferation and synergy assays

For proliferation assays, cells were plated in 6-well plates (Corning), and growth was measured using daily or twice daily brightfield imaging and cell counting with the Celigo Imaging Cytometer (Nexcelom). Media was exchanged for fresh media with drugs every 3–4 days. Cell nuclei were also counted at the endpoint using fluorescence imaging of cells stained with 5 µg/mL Hoechst 33342 (Sigma–Aldrich) in PBS. Statistical analyses were performed using GraphPad Prism. For synergy studies, experiments were performed in 384-well plates (Corning). SUM159 cells were seeded at a density of 200 cells/well, SUM159R and SUM149 at a density of 500 cells/well, and SUM149R at a density of 1000 cells/well, in 50 µL of media. The following day, drugs were pin-transferred into the wells using the JANUS Automated Workstation (Perkin Elmer), from a 500X concentrated drug plate made in a 384-well plate (Greiner Bio-One). Five concentrations for each drug were chosen to achieve between 20% and 80% inhibition, with four replicate wells per concentration of each drug alone, and half doses were used for combination treatments, with eight replicate wells per concentration pair. After 3 days, cells were stained with 5 µg/mL Hoechst 33342 in PBS, and nuclei were imaged and counted using the Celigo Imaging Cytometer. Statistical analyses were performed using R. Combination indices were calculated using the Chou-Talalay method^51^.

### Lentiviral infection of barcodes and reporter constructs

The high-complexity ClonTracer barcode library was a gift from Frank Stegmeier (Novartis). 100 million SUM159 cells were barcoded by lentiviral infection using 8 µg/mL Polybrene (Millipore) as described^20^. After 24 h of incubation with virus, infected cells were selected with 2 μg/mL puromycin. To ensure that the majority of cells were labeled with a single barcode per cell, we used a target m.o.i. of approximately 0.1, corresponding to 10% infectivity after puromycin selection. Infected cell populations were expanded in culture for the minimal amount of time to obtain a sufficient number of cells to set up replicate experiments.

For ploidy studies, SUM159, SUM149, CAL-51, and MCF10A cells were labeled with the FastFUCCI reporter (pBOB-EF1-FastFUCCI-Puro, AddGene). SUM159 cells were also labeled with GFP (pEGFP-N1, Clontech) and RFP (pDsRed-Monomer-C1, Clontech) for fusion assays. For all virus production, 293FT cells were transfected with plasmid, TransIT-293 Reagent Transfection Reagent (Mirus Bio), and Ready-to-Use Packaging Plasmid Mix (Cellecta). Transfection media was changed the next day. Media with virus particles was collected 2–3 days later and passed through a 0.45 µm syringe filter followed by virus concentration using Lenti-X Concentrator (Takara) as directed. Concentrated virus was added to a 10 cm dish of 70% confluent cells with 8 µg/mL Polybrene (Millipore). FastFUCCI-labeled cells were selected with 1 µg/mL puromycin and expanded for 6–9 days and then sorted by FACS for GFP^+^ or mCherry^+^ cells. SUM159-GFP and RFP cells were sorted for positive cells. Tetraploid-enriched lines were generated by staining SUM159-FUCCI cells with 10 µg/mL Hoechst 33342 (Sigma–Aldrich) and sorting for GFP^+^/mCherry^−^/Hoechst^high^ cells, and by staining co-cultured SUM159-GFP and RFP cells with Hoechst 33342 and sorting for GFP^+^/RFP^+^ cells.

For time-lapse live cell imaging, SUM159 cells were first infected with H2B-GFP (H2B-GFP, Addgene, subcloned into pLenti5/V5, Invitrogen) and sorted for GFP^high^ cells. These cells were then expanded and infected with membrane-TdTomato (pQC membrane TdTomato-IX, Addgene, subcloned into pLenti5/V5, Invitrogen). GFP^+^/TdTomato^low^ cells were sorted, expanded, and then sorted again for GFP^+^/TdTomato^+^ cells.

### CRISPR knockout

CRISPR guides targeting *RB1* were designed using CRISPOR^52^: (1) TCCTGAGGAGGACCCAGAGC, (2) CGGTGGCGGCCGTTTTTCGG, (3) GGACAGGGTTGTGTCGAAAT. Guides were cloned in lentiCRISPRv2 as previously described^53^. For virus production HEK293T cells were transfected with the respective lentiCRISPRv2-plasmid (empty vector served as control) and lentiviral envelope (pCMV-VSV-G) and packaging (pCMV-Δ8.9) plasmids using PolyFect (Qiagen). Transfection media was changed the next day. Media with virus particles was collected 48 hours later and passed through a 0.45 µm syringe filter followed by virus concentration using Amicon Ultra-15 100kDa centrifugal columns. Concentrated virus was added to 8 × 10^4^ SUM159 cells together with 10 µg/mL Polybrene (Millipore). To achieve efficient *RB1* knockout, virus from all three guides was used on the same cells. The same amount of virus carrying empty lentiCRISPRv2 was used for control cells. 48 hours post-infection, media was changed, and cells were placed under puromycin selection (3 µg/mL) for 6 days.

### siRNA knockdown

SUM159 and CAL-51 were transfected with CDK4 (Assay ID s2822), CDK6 (Assay ID s51), or scramble (Silencer Select, Negative Control #1) siRNA (Thermo Fisher Scientific) using Lipofectamine RNAiMAX (Life Technologies) following the manufacturer’s instructions with a final siRNA concentration of 10 nM.

### Flow cytometry analysis

For cell cycle analysis, cells were fixed overnight in 70% ethanol and then stained in 20 µg/mL propidium iodide (Thermo Fisher Scientific) with 0.2 mg/mL PureLink RNase A (Thermo Fisher Scientific) in 0.1% Triton X-100 (Sigma–Aldrich) for 30 min. For analysis of apoptosis, cells were stained using the Alexa Fluor 488 Annexin V/Dead Cell Apoptosis Kit as directed (Thermo Fisher Scientific). For ploidy studies, cells were stained with PBS with 10 µg/mL Hoechst 33342 (Sigma–Aldrich), 0.5% NP-40, and 3.7% paraformaldehyde overnight at 4 °C or with 7-AAD according to manufacturer’s instructions. Fluorescence intensities were acquired on an LSRFortessa cytometer (BD Biosciences). Data were analyzed using FlowJo or Cytobank.

### Xenograft studies

Barcoded SUM159 cells and SUM159R cells were injected orthotopically into the mammary fat pads of 6-week-old female NOG (Taconic) or NSG mice (Jackson Laboratory). Two million cells were injected into each fat pad in 25 µL of DMEM/F12 and 25 µL of Matrigel (Corning). Once tumors became palpable, mice were randomized with five mice per group and treated for up to 2 weeks. 1:9 DMSO in hydroxypropyl-β-cyclodextrin (Sigma–Aldrich) was used as vehicle and administered daily by intraperitoneal (i.p.) injection. JQ1 was dosed at 30–50 mg/kg daily i.p., palbociclib at 75 mg/kg daily by gavage, and paclitaxel at 10 mg/kg twice weekly i.p. For drug schedule studies, mice were treated with vehicle for 2 weeks, JQ1 for 1 week followed by paclitaxel for 1 week, paclitaxel for 1 week followed by JQ1 for 1 week, or JQ1 + paclitaxel for 1 week followed by vehicle for 1 week.

Mouse body weights and caliper measurements of tumor size were recorded every 3 days. After completion of treatment, mice were euthanized, and tumors were dissected and fixed in formalin and submitted to the Brigham and Women’s Pathology Core for paraffin embedding or flash frozen for further study. Statistical analyses were performed using GraphPad Prism.

### Histology, immunofluorescence, and immunoblotting

FFPE xenografts chunks were cut into slides and stained with hematoxylin and eosin (H&E) by the Brigham and Women’s Hospital Pathology Core Facility. For immunofluorescence, unstained slides were deparaffinized, and antigen retrieval was performed in Dako Target Retrieval Solution pH 9 (Agilent) for 30 min in a steamer. Slides were blocked for 10 min with 10% goat serum and then stained with anti-cyclin D1 primary antibody (1:50, Abcam, ab134175) at 4 °C overnight, followed by staining with anti-rabbit secondary antibody (1:100, Thermo Fisher Scientific, A21245) for 30 min at room temperature. Slides were then incubated with primary antibodies against cleaved caspase 3 (1:100, Cell Signaling Technology, 9661) and phospho-histone H3 (1:400, Abcam, ab5176), labeled with Zenon Alexa Fluor 488 and 555 Rabbit IgG Labeling Kits, respectively (Thermo Fisher Scientific, Z25302 and Z25305), for 1 h at room temperature.

For immunofluorescence on in vitro experiments, cells were grown on glass cover slips. After treatment with experimental compounds, cells were fixed in methanol for 5 min, then washed with PBS and blocked with 10% goat serum for 1 h. Cells were stained with primary antibodies against phospho-histone H3 (1:400, Abcam, ab5176) and α-tubulin (1:100, Sigma–Aldrich, T9026) at 4 °C overnight, followed by staining with anti-rabbit (1:100, Thermo Fisher Scientific, A11008) and anti-mouse IgG1 (1:100, Thermo Fisher Scientific, A21125) secondary antibodies for 1 h at room temperature. All slides were imaged on a Leica SP5X laser scanning confocal microscope.

For immunoblotting, cells were lysed in RIPA buffer with protease and phosphatase inhibitors and sonicated in a Covaris sonicator for 5 min. Lysates were then denatured in NuPAGE LDS Sample Buffer 4X (Thermo Fisher Scientific) and DTT at 70 °C for 10 min. Proteins were resolved with NuPAGE 4–12% Bis-Tris gels (Thermo Fisher Scientific) in NuPAGE MOPS SDS Running Buffer (Thermo Fisher Scientific) and transferred to PVDF membranes in NuPAGE Transfer Buffer (Thermo Fisher Scientific). Membranes were blocked in 5% milk in TBST for 1 h at room temperature and incubated with primary antibodies at 4 °C overnight: pRb S780 (Cell Signaling Technology, 8180), CDK4 (Thermo Fisher Scientific, MS616P1), CDK6 (Abcam, ab54576), β-actin (Sigma–Aldrich, A2228), GAPDH (Cell Signaling Technology, 5174). Membranes were then incubated with HRP anti-rabbit (Fisher Scientific, 656120) and anti-mouse (Thermo Fisher Scientific, 32430) secondary antibodies for 1 h at room temperature. Chemiluminescence detection was performed using Pierce ECL Western Blotting Substrate (Thermo Fisher Scientific).

### Clonal selection with JQ1 combinations in vitro

Barcoded SUM159 cells were grown in DMSO, JQ1 (100 nM), paclitaxel (0.6 nM), palbociclib (160 nM), JQ1 + paclitaxel, or JQ1 + palbociclib, in triplicates. Cells were initially plated in 10 cm plates (Falcon) with 2 million cells per plate and then split 1:4 when approximately 80% confluent. The remaining cells at each passage were frozen viably for further analysis. Cells were grown for up to 18 passages.

### Mathematical modeling of barcode selection

We used a birth-death process to model the selection of clones, comprising two cell types: sensitive cells, with an initial proportion of 1-*ρ*, and resistant cells, with an initial proportion *ρ*. Untreated and treated sensitive and resistant cells have individual birth rates ($b_{{\mathrm{s}}}^j$ and $b_{{\mathrm{r}}}^j$) and death rates ($d_{{\mathrm{s}}}^j$ and $d_{{\mathrm{r}}}^j$), which are log normally distributed and where *j* = DMSO or drug (JQ1 or palbociclib). For instance, $b_{{\mathrm{s}}}^{{\mathrm{drug}}}$ is the birth rate of sensitive cells under drug treatment. The means of the growth rates ($\lambda _i^j = b_i^j - d_i^j$, where *i* = s or r) were measured from proliferation assays of pre-treatment cells and resistant lines under DMSO, JQ1, or palbociclib treatment. Resistant lines comprised mixtures of 10 single-cell clones from the last passage of each of the three post-drug selection replicates, derived from growing sparsely plated cells in the presence of JQ1 or palbociclib. The growth rates of the pre-treatment cells was considered to be a combination of the growth rates of both sensitive and pre-existing resistant cells. Assuming a small enough growth rate such that the contribution of de novo resistant cells to the population at the end of the growth assay is negligible, the number of cells can be approximated as the sum of the individual growth rates, $X^j\left( t \right) \approx \rho X^j\left( 0 \right)e^{\lambda _{{\mathrm{r}}}^jt} + \left( {1 - \rho } \right)X^j\left( 0 \right)e^{\lambda _{{\mathrm{s}}}^jt}$, where *X*^*j*^(*t*) is the expected number of cells in condition *j* at time *t*. Thus, the growth rates of sensitive cells are estimated as

1 $$\lambda _{{\mathrm{s}}}^j \approx \frac{1}{t}\log \frac{{X^j\left( t \right) - \rho X^j\left( 0 \right)e^{\lambda _{{\mathrm{r}}}^jt}}}{{\left( {1 - \rho } \right)X^j\left( 0 \right)}}$$

using the duration of the proliferation assays as *t*. Death rates were estimated from flow cytometry data of percentages of apoptotic cells after treatment of pre-treatment cells with DMSO, JQ1, or palbociclib as follows. Assuming a small proportion of resistant cells and a small death rate, the expected number of cells in condition *j* at time *t* was estimated to be $X^j\left( t \right) \approx X^j(0)e^{\lambda _{{\mathrm{s}}}^jt}$. Thus, the number of cells that will die in the next infinitesimal time step, *τ*, is

2 $$d_{{\mathrm{s}}}^jX^j\left( \tau \right) \approx d_{{\mathrm{s}}}^jX^j(0)e^{\lambda _{{\mathrm{s}}}^j\tau }$$

so the expected number of cells in condition *j* that have died up to time *t* is

3 $$D^j\left( t \right) \approx \mathop {\smallint }\limits_0^{\,\,\,\,t} d_s^jX^j(0)e^{\lambda _s^j\tau }d\tau = \frac{{d_s^jX^j(0)}}{{\lambda _s^j}}(e^{\lambda _s^j\tau } - 1)$$

Therefore, the fraction of dead cells by flow cytometry at time *t* is estimated as

4 $$\frac{{D^j(t)}}{{X^j\left( t \right) + D^j(t)}} \approx \frac{{d_{{\mathrm{s}}}^j(e^{\lambda _{{\mathrm{s}}}^jt} - 1)}}{{e^{\lambda _{{\mathrm{s}}}^jt}\left( {\lambda _{{\mathrm{s}}}^j + d_{{\mathrm{s}}}^j} \right) - d_{{\mathrm{s}}}^j}}$$

from which we then calculated the death rate of sensitive cells, $d_{{\mathrm{s}}}^j$. The death rates of resistant cells under treatment, $d_{{\mathrm{r}}}^{{\mathrm{drug}}}$, were assumed to be equivalent to that of untreated sensitive cells, $d_{{\mathrm{s}}}^{{\mathrm{DMSO}}}$. Birth rates were thus calculated as $b_i^j = \lambda _i^j + d_i^j$.

The initial barcode distribution was determined from the sequencing results. Across all libraries sequenced, we observed 903,900 unique barcodes. Thus, we estimated the complexity of the entire library to be ~1 million. We used the combined barcode frequency observed in three pre-treatment sequencing libraries to estimate the initial barcode distribution. In these three libraries, we observed a total of 294,844 unique barcodes, whose frequencies were used to estimate the distribution of those top barcodes. The frequencies of each of the remaining 705,156 barcodes were assumed be less than the minimum barcode frequency observed in the three pre-treatment libraries and were drawn from an exponential distribution. For each barcode, we sampled a birth and death rate from a log normal distribution centered around the experimentally derived parameters and with a variance of 10% of the means. These birth and death rates were heritable to all daughter cells. To simulate plating, we sampled three replicate plates of 2 million cells each from a starting pool of 700 million cells with this estimated pre-treatment distribution.

To simulate growth and mutation during passaging in drug, we used the Binomial-Negative Binomial (BNB) algorithm^54^. For resistant cells, which do not undergo further mutation, expansion is simulated for one time step Δ*t* as the sum of *m* ~ *B*(*n*_0_, 1 − *α*) and *n* ~ *NB*(*m*, 1 − *β*), where

5 $$\alpha ({\mathrm{\Delta }}t) = \frac{{d_{{\mathrm{r}}}^{{\mathrm{drug}}}e^{(b_{{\mathrm{r}}}^{{\mathrm{drug}}} - d_{{\mathrm{r}}}^{{\mathrm{drug}}})\Delta t} - d_{{\mathrm{r}}}^{{\mathrm{drug}}}}}{{b_{{\mathrm{r}}}^{{\mathrm{drug}}}e^{(b_{{\mathrm{r}}}^{{\mathrm{drug}}} - d_{{\mathrm{r}}}^{{\mathrm{drug}}})\Delta t} - d_{{\mathrm{r}}}^{{\mathrm{drug}}}}}\,{\mathrm{and}}\,\beta ({\mathrm{\Delta }}t) = \frac{{b_{{\mathrm{r}}}^{{\mathrm{drug}}}e^{(b_{{\mathrm{r}}}^{{\mathrm{drug}}} - d_{{\mathrm{r}}}^{{\mathrm{drug}}})\Delta t} - b_{{\mathrm{r}}}^{{\mathrm{drug}}}}}{{b_{{\mathrm{r}}}^{{\mathrm{drug}}}e^{(b_{{\mathrm{r}}}^{{\mathrm{drug}}} - d_{{\mathrm{r}}}^{{\mathrm{drug}}})\Delta t} - d_{{\mathrm{r}}}^{{\mathrm{drug}}}}}$$

according to^55^. The binomially distributed variable *m* simulates the number of starting cells that do not go extinct within Δ*t*, while the negative binomially distributed variable *n* simulates the proliferation of those *m* cells.

For sensitive cells, the birth rate is adjusted for mutation, which occurs at rate *μ*, so that the adjusted birth rate is $b_{{\mathrm{s}}}^{{\mathrm{drug}} \ast } = b_{{\mathrm{s}}}^{{\mathrm{drug}}}(1 - \mu )$ and the mutation rate is $\mu ^ \ast = \mu b_{{\mathrm{s}}}^{{\mathrm{drug}}}$. Following the BNB algorithm, in each time step, we sampled *r* ~ *U*(0, 1) for each sensitive barcode to sample the next mutation time

6 $$t_m = \frac{1}{R}\log \left( {\frac{{r^{1/n_0}\left( {R - W + 2b_{{\mathrm{s}}}^{{\mathrm{drug}} \ast }} \right) - W - R + 2d_{{\mathrm{s}}}^{{\mathrm{drug}}}}}{{r^{1/n_0}\left( { - R - W + 2b_{{\mathrm{s}}}^{{\mathrm{drug}} \ast }} \right) - W + R + 2d_{{\mathrm{s}}}^{{\mathrm{drug}}}}}} \right)$$

and the next extinction time

7 $$t_e = \frac{1}{R}\log \left( {\frac{{W - R - 2d_{{\mathrm{s}}}^{{\mathrm{drug}}}r^{-1/n_0}}}{{W + R - 2d_{{\mathrm{s}}}^{{\mathrm{drug}}}r^{-1/n_0}}}} \right)$$

where $R = \sqrt {(b_{{\mathrm{s}}}^{{\mathrm{drug}} \ast } - d_{{\mathrm{s}}}^{{\mathrm{drug}}})^2 + (2b_{{\mathrm{s}}}^{{\mathrm{drug}} \ast } + 2d_{{\mathrm{s}}}^{{\mathrm{drug}}} + \mu ^ \ast )\mu ^ \ast }$, $W = b_{{\mathrm{s}}}^{{\mathrm{drug}} \ast } + d_{{\mathrm{s}}}^{{\mathrm{drug}}} \,+ \mu ^ \ast$, and *n*_0_ is the starting number of sensitive cells of that barcode. Thus, barcodes that go extinct have

8 $$r \, < \, \left( {\frac{{R - W + 2d_{{\mathrm{s}}}^{{\mathrm{drug}}}}}{{R + W - 2b_{{\mathrm{s}}}^{{\mathrm{drug}} \ast }}}} \right)^{n_0}$$

Barcodes that go extinct within the next time step, i.e. *t*_*e*_ < Δ*t*, are replaced with a count of 0. We used Δ*t* = 1 day, since in the experiment, cells were checked once per day and split if they reached 80% confluence. Expansion of barcodes that did not mutate within the time step, i.e., *t*_*m*_ > Δ*t*, was simulated according to Eq. (5) with modifications such that $n \, \ne \, 0$. Then, for each mutation event occurring within Δ*t*, the expansion of sensitive cells of that barcode is simulated up to the mutation time *t*_*m*_ as the sum of $\tilde m\sim B\left( {n_0 - 1,1 - p_E\left( {t_m} \right)/p_M\left( {t_m} \right)} \right)$, 1, and $\tilde n\sim NB\left( {\tilde m + 2,p_B\left( {t_m} \right)} \right)$, where

9 $$\begin{array}{l}p_M\left( {t_m} \right) = \frac{{RC\left( {t_m} \right) \, + \, 2d_{{\mathrm{s}}}^{{\mathrm{drug}}}S\left( {t_m} \right) \, - \, WS(t_m)}}{{RC\left( {t_m} \right) \, - \, 2b_{{\mathrm{s}}}^{{\mathrm{drug}} \ast }S\left( {t_m} \right) \, + \, WS(t_m)}}\\ p_E\left( {t_m} \right) = \frac{{d_{{\mathrm{s}}}^{{\mathrm{drug}}}(1 \,- \, p_M\left( {t_m} \right))}}{{W \, -\, d_{{\mathrm{s}}}^{{\mathrm{drug}}} \, - \, b_{{\mathrm{s}}}^{{\mathrm{drug}} \ast }p_M\left( {t_m} \right)}}\hfill \\ p_B\left( {t_m} \right) = \frac{{b_{{\mathrm{s}}}^{{\mathrm{drug}} \ast }p_E\left( {t_m} \right)}}{{d_{{\mathrm{s}}}^{{\mathrm{drug}}}}}\hfill\end{array}$$

and where $C\left( t \right) = \cosh \frac{{Rt}}{2}$ and $S\left( t \right) = \sinh \frac{{Rt}}{2}$. One resistant cell of that barcode is added, and its expansion for the rest of the time step is simulated according to Eq. (5). A new mutation time is then simulated for that barcode according to Eqs. (6)–(8). Expansion of other barcodes up to *t*_*m*_ is simulated according to Eq. (5), with modifications such that $n \, \ne \, 0$. These steps are repeated until all next mutation times are beyond ∆*t*.

The simulation is allowed to run for another ∆*t*, until the total number of cells reaches a number corresponding to 80% confluence (5 million). A split of 1:4 is then simulated by sampling down to ¼ of the population size. If the current passage is a passage for which we performed barcode sequencing in the experiment, the other ¾ of the cells are outputted for diversity analysis. The entire BNB algorithm is repeated for a total of 18 passages.

The simulation was run for a range of parameters *ρ* and *μ*. A wide range from *ρ* = 1 × 10^−1^ to 1 × 10^−6^ and from *μ* = 1 × 10^−2^ to 1 × 10^−6^ was first sampled for both JQ1 and palbociclib selection with one simulation run for each parameter combination. A smaller range that more closely matched the experimental data was then chosen and sampled, with five simulation runs for each parameter combination.

### DNA/RNA extraction

DNA and RNA were extracted from cultured cells and xenografts using the AllPrep DNA/RNA Mini Kit (Qiagen) as directed. Viably frozen cells from the last treatment passage were thawed and treated for 2–3 additional passages before DNA/RNA extraction. Frozen tumors were pulverized using the Covaris CP02 Tissue Pulverizer. Tissues or cells were homogenized by passing the sample through a 23g needle. Isolated RNA was treated with the RNase-Free DNase Set (Qiagen). RNA was prepared from duplicate samples. The QIAamp DNA Maxi and Mini Kits (Qiagen) were also used to extract DNA from those samples on which we did not perform RNA-seq.

### Barcode sequencing

PCR was used to amplify barcodes and introduce Illumina adaptors along with a 5 bp index sequence for multiplexing as described^20^. Two microgram of genomic DNA was used as template, and 15–16 samples were multiplexed for NGS. PCR products were run on 0.8% agarose NGS E-Gels (Invitrogen) to verify the correct library size, and bands were cut out and purified using the MinElute Gel Extraction Kit (Qiagen) as directed.

### RNA-sequencing

Bulk RNA-seq libraries were prepared from total RNA by the Dana-Farber Cancer Institute Molecular Biology Core Facilities (MBCF) using the Illumina TruSeq Stranded mRNA Library Prep Kit, and 16–18 samples were multiplexed per lane for NGS. For single-cell RNA-seq, equal numbers of cells from triplicates were pooled and washed twice in PBS with 0.04% RNase-free BSA (New England BioLabs). Cells were then diluted to 700 cells/µL and filtered through a 35 µm nylon mesh prior to library preparation. Single-cell RNA-seq libraries were prepared using the Chromium Single Cell 3’ Library & Gel Bead Kit v2 and Single Cell A Chip (10X Genomics) as directed. Samples were multiplexed 8 per lane for NGS. MBCF performed all sequencing.

### Whole-exome sequencing

Libraries were prepared from equal amounts of pooled genomic DNA from triplicates by the Dana-Farber Cancer Institute Center for Cancer Genome Discovery (CCGD). Prior to library preparation, DNA was fragmented to 250 bp (Covaris ultrasonication) and further purified using Agencourt AMPure XP beads. Size-selected DNA was ligated to sequencing adaptors with sample-specific barcodes using the KAPA Hyper Prep Kit. Libraries were pooled and sequenced on an Illumina MiSeq nano flow cell to estimate the library DNA concentration based on the number of barcode reads per sample. Libraries were pooled and captured using the SureSelectXT Human All Exon v5 (Agilent) in 7 × 3-plex and 1 × 2-plex. Captures were performed using the SureSelectXT Reagent Kit (Agilent). Captures were pooled together and sequenced on four lanes of the Illumina HiSeq 3000.

### Karyotyping

Karyotyping was performed by the Brigham and Women’s Hospital CytoGenomics Core. Twenty metaphases were counted from each treatment sample and 5–6 cells were karyotyped.

### Metaphase spreads

Cells were treated overnight with 0.4 µg/mL colcemid. Harvested cells were then treated with 75 mM KCl for 10–20 min and washed three times in 3:1 methanol/acetic acid fixative. Cells resuspended in fixative were dropped onto slides and allowed to dry. Slides were then stained with DAPI.

### Droplet digital PCR (ddPCR)

Taqman primer/probe mix was custom-made by Life Technologies. The allele-specific MGB probes were labeled with either VIC or FAM at the 5′ end and a nonfluorescent quencher (NFQ) at the 3′ end. The forward primer sequence was 5ʼ-ACAGCGACCGTGTGCTC-3′, reverse primer sequence was 5′-TTCAGTGGTTTAGGAGGGTTGC-3′, wild-type (E864) probe sequence was 5′-VIC-AAGAAGTGCTGAAGGAA-MGB-NFQ-3′, and mutant (E864*) probe sequence was 5′-FAM-AAAAGAAGTGCTTAAGGAA-MGB-NFQ-3′. ddPCR cycling conditions were: 95 °C for 10 min, 40 cycles of 94 °C for 30 seconds, and 57 °C for 60 seconds, 10 °C forever. The reaction mixture (25 uL) included ddPCR Supermix for Probes (Bio-Rad), custom-made Taqman primer/probe mix, and appropriate DNA templates. Droplets were generated on the Automated Droplet Generator (Bio-Rad). Reactions were cycled on a thermocycler and were read on the QX200 Droplet Reader (Bio-Rad). Data analysis was performed with QuantaSoft (Bio-Rad).

### Time-lapse live cell imaging

SUM159 cells with H2B-GFP/membrane-TdTomato were plated in 24-well µ-plates (ibidi) with 20,000 cells per well. Starting the following day, images were collected every 10 min for 48 h from 10–15 positions in each well, immediately after addition of fresh media with DMSO, JQ1, palbociclib, or JQ1 + palbociclib. Imaging dishes were mounted on a TE2000-E2 inverted microscope equipped with a Nikon Perfect Focus system. Images were acquired from Andor brightfield, FITC, and Cy3 channels from 4 z-steps of 0.5 µm, using a 20X 0.5 NA Plan Fluor objective. An Okolab cage incubator was used to maintain samples at 37 °C and 5% humidified CO_2_. Image acquisition was controlled with MetaMorph (Molecular Devices). For data analysis, the lengths of each phase of the cell cycle were quantified for the duration of the live imaging period (*n* ≈ 35 cells for each treatment condition starting from the images at the first time point). Nuclear condensation was defined as the beginning of prometaphase, chromosomal segregation as the beginning of anaphase, furrow ingression as the beginning of cytokinesis, and nuclear decondensation and reattachment of cells as the beginning of interphase. After each cell division, only one daughter cell was analyzed in subsequent cell cycles.

### Barcode analysis

Barcode sequencing reads were demultiplexed and filtered for reads with 30 bp length, containing an Illumina adaptor, matching the barcode pattern (alternating A/T and C/G), having a Phred quality score of at least 10 for all base pairs and an average Phred score of 30. For each sample, barcodes that had only 1 read were also filtered out. Changes in barcode diversity over time and between replicates were visualized using the fishplot R package^56^. The Shannon index was used to quantify barcode diversity and is given by

10 $$H = - \sum_i p_i\ln p_i$$

where *p*_*i*_ is the frequency for barcode *i*. Because of the high complexity of the barcode library, we limited our analysis to barcodes that were observed in at least one sequencing library of the plates being compared, to ensure that we were comparing barcodes that were present in the initial seeded pools. To compare replicates within treatment groups, we considered the intersection between replicate plates of the unions of barcodes observed across all passages sequenced. When comparing between treatment groups, we used the intersection of barcodes seen in all replicate plates in all treatments in at least one passage (*n* = 13,248 for JQ1 + palbociclib experiment and 16,330 for JQ1 + paclitaxel experiment).

### Validation of mathematical model of barcode selection

Simulation results were compared with experimental data using the Shannon index and the proportion of overlapping barcodes. To determine the best-fit parameters, we calculated a likelihood score for each parameter set (*ρ*, *μ*) using the distribution of proportions of shared barcodes over five independent runs of the simulation. This was computed as the sum of the likelihoods of observing the proportion of shared barcodes *x*_*i,j,k*_ between *i* = 1, 2, and 3 replicates, assuming a normal distribution with mean *μ*_*ρ*,*μ*,*i*_ and variance $\sigma _{\rho ,\mu ,i}^2$ seen over the five simulations using parameters (*ρ*, *μ*), for each number of top barcodes *j* in each replicate *k*, summed over the three experimental replicates,

11 $${\cal{L}}\left( {\rho ,\mu {\mathrm{|}}x} \right) = \mathop {\sum }\limits_{\begin{array}{*{20}{c}} {i\!=\!1,2,3;} \\ { j;k \!=\!1,2,3} \end{array}} \left( {2\pi \sigma _{\rho ,\mu ,i,j}^2} \right)^{ - 1/2}{\mathrm{exp}}\left( { - \frac{1}{2}\frac{{(x_{i,j,k} - \mu _{\rho ,\mu ,i,j})^2}}{{\sigma _{\rho ,\mu ,i,j}^2}}} \right)$$

### RNA-seq analysis

RNA-seq reads were aligned to the hg19 build of the human genome using STAR v2.6.0a^57^ and counted using HTSeq-count v0.6.1p1^58^. Libraries were then normalized for sequencing depth, and differential genes were determined using DESeq2 v1.20.0^59^. Differential gene lists (genes with *p*_adj_ < 0.05) were analyzed for enriched pathways and process networks using MetaCore v19.4, using a cutoff of FDR < 0.05 as significantly enriched.

### Single-cell RNA-seq analysis

Single-cell RNA-seq reads were aligned to the hg19 build of the human genome and counted using CellRanger v2.1.0 (10X Genomics). Using Seurat v2.3.4^60^, cells with a low or high level of mitochondrial genes (less than 1% or greater than 15%) or a low number of detected genes (less than 2000) were filtered out. We only considered genes expressed in a minimum of three cells. Gene counts were log-normalized and scaled, and the percent of mitochondrial genes and number of UMIs were regressed out. Cell cycle scoring was performed according to^61^ and the difference between the S and G2M scores was also regressed out. The top principal components were then calculated and used to perform clustering with a resolution of 0.4 and *t*-Distributed Stochastic Neighbor Embedding (*t*-SNE) analysis. Differential genes for each cluster were identified (genes with *p*_adj_ < 0.05), and differential gene lists were analyzed for enriched pathways and process networks using MetaCore, using a cutoff of FDR < 0.05 as significantly enriched.

### Exome analysis

Exome data were analyzed by the Dana-Farber Cancer Institute Center for Cancer Genome Discovery (CCGD). Reads were demultiplexed and aligned to the reference sequence b37 edition from the Human Genome Reference Consortium using Picard (http://broadinstitute.github.io/picard). Mutation analysis for single nucleotide variants (SNV) was performed using MuTect v1.1.4^62^ and annotated by Variant Effect Predictor (VEP) v79^63^. After initial identification of SNVs and indels by MuTect and GATK v1.65, respectively, the variants were annotated using OncoAnnotate v1.3.0 to determine what genes are impacted and their effect on the amino acid sequence.

Copy number variants were identified using RobustCNV, an algorithm in development by the CCGD. Briefly, RobustCNV relies on localized changes in mapping depth of sequenced reads in order to identify changes in copy number. Observed values are normalized against the mapping depth in a panel of normals (PON) sampled with the same capture bait set. Normalized coverage data are then segmented using Circular Binary Segmentation^64^ with the DNAcopy Bioconductor package. Finally, segments are assigned gain, loss, or normal-copy calls using a cutoff derived from the within-segment standard deviation and a tuning parameter set based on comparisons to array-CGH calls in separate validation experiments.

### Reporting summary

Further information on research design is available in the Nature Research Reporting Summary linked to this article.

## Supplementary information

Supplementary Information

Description of Additional Supplementary Files

Supplementary Data 1

Supplementary Data 2

Supplementary Data 3

Supplementary Movie 1

Supplementary Movie 2

Supplementary Movie 3

Supplementary Movie 4

Supplementary Movie 5

Supplementary Movie 6

Reporting Summary

## Data Availability

The barcode, exome, RNA-seq, and single-cell RNA-seq data have been deposited in GEO under accession number GSE131986. The source data underlying Fig. 1a–b, f, 3b, 4, 7, and Supplementary Fig. 7h–i are provided as a Source Data file.

## Source data

Source data
Supplementary Mathematical Model Code

## Code availability

The source code for the mathematical modeling is available within the Supplementary Information files.
