# Supplementary material for: Acquired resistance to combined BET and CDK4/6 inhibition in triple-negative breast cancer
Source: Nat Commun. 2020 May 11;11:2350. doi: 10.1038/s41467-020-16170-3 (PMC7214447; doi:10.1038/s41467-020-16170-3)
Supplement: Supplementary file 1 — Supplementary Information [file 41467_2020_16170_MOESM1_ESM.pdf]

Supplementary Information

Acquired resistance to combined BET and CDK4/6 inhibition in triple-negative breast cancer

Ge et al.

SUPPLEMENTARY FIGURES

Supplementary Figure 1. Flow cytometry analysis of cell cycle and apoptosis. a, Gating strategy for flow cytometry analysis of cell cycle in PI-stained cells (shown are DMSO-treated cells). **b**, Representative histograms of PI-stained SUM159 cells in each cell cycle phase, following 24 hours of treatment with the indicated compounds (PAL, palbociclib, and TAX, paclitaxel), by flow cytometry fitted to the Watson cell cycle model. **c**, Representative scatterplots of SUM159 cells that are viable (annexin V-/PI-), early apoptotic (annexin V+/PI-), and late apoptotic (annexin V+/PI+), using flow cytometry following three days of treatment.

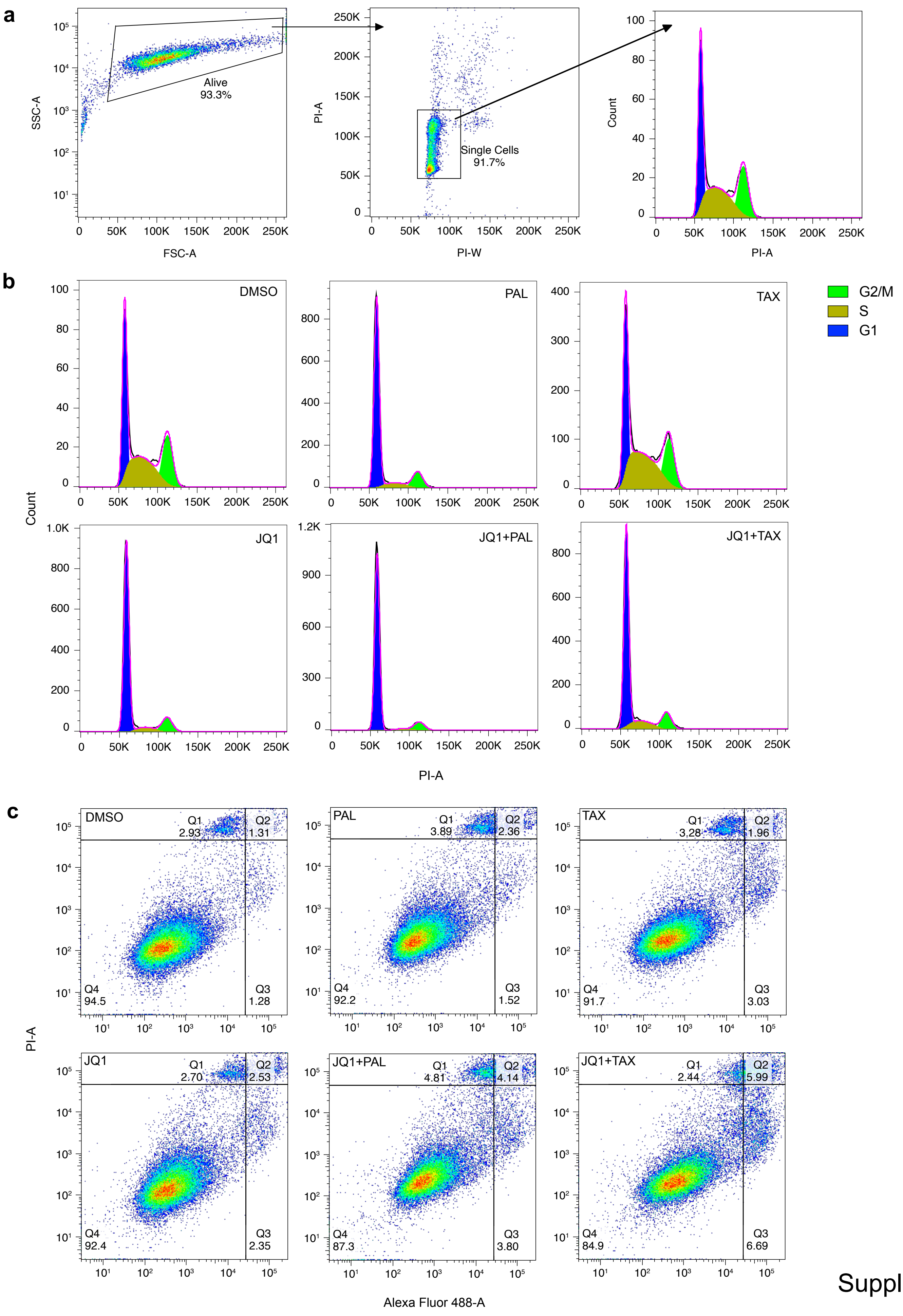

a
SSC-A
FSC-A
Alive
93.3%
PI-A
PI-W
Single Cells
91.7%
Count
PI-A
b
DMSO
PAL
TAX
JQ1
JQ1+PAL
JQ1+TAX
G2/M
S
G1
Count
PI-A
c
DMSO
Q1 2.93
Q2 1.31
Q4 94.5
Q3 1.28
PAL
Q1 3.89
Q2 2.36
Q4 92.2
Q3 1.52
TAX
Q1 3.28
Q2 1.96
Q4 91.7
Q3 3.03
JQ1
Q1 2.70
Q2 2.53
Q4 92.4
Q3 2.35
JQ1+PAL
Q1 4.81
Q2 4.14
Q4 87.3
Q3 3.80
JQ1+TAX
Q1 2.44
Q2 5.99
Q4 84.9
Q3 6.69
PI-A
Alexa Fluor 488-A

Suppl Fig 1

Supplementary Figure 2. Barcode diversity in xenografts and cells treated with JQ1 combinations. **a**, Cumulative frequencies of ranked barcodes in SUM159 xenografts treated for 2 weeks with JQ1 and palbociclib (left) or JQ1 and paclitaxel (right) alone and in combination. **b**, Scatterplots of Shannon indices vs. tumor weights of xenografts following 2 weeks of treatment with JQ1 and palbociclib (left), before treatment and following 1 week of treatment with JQ1 and paclitaxel (center), and following 2 weeks of treatment with JQ1 and paclitaxel, alone and in combinations. **c**, Venn diagram of the number of shared barcodes between four xenografts prior to treatment. **d**, Cumulative frequencies of ranked barcodes in SUM159 cells at various passages of treatment with DMSO, JQ1, palbociclib (PAL), and JQ1+palbociclib (JQ1+PAL). **e**, Cumulative frequencies of ranked barcodes in SUM159 cells at various passages of treatment with DMSO, JQ1, paclitaxel (TAX), and JQ1+paclitaxel. **f**, Frequencies of top barcodes (those representing at least 0.5% of the population at any passage) in cell populations selected with JQ1 and paclitaxel, alone and in combination. Colors represent unique barcodes, gray background represents all other barcodes in the population, and each plot represents one replicate. Frequencies of all barcodes (*y*-axis) add up to 100%.

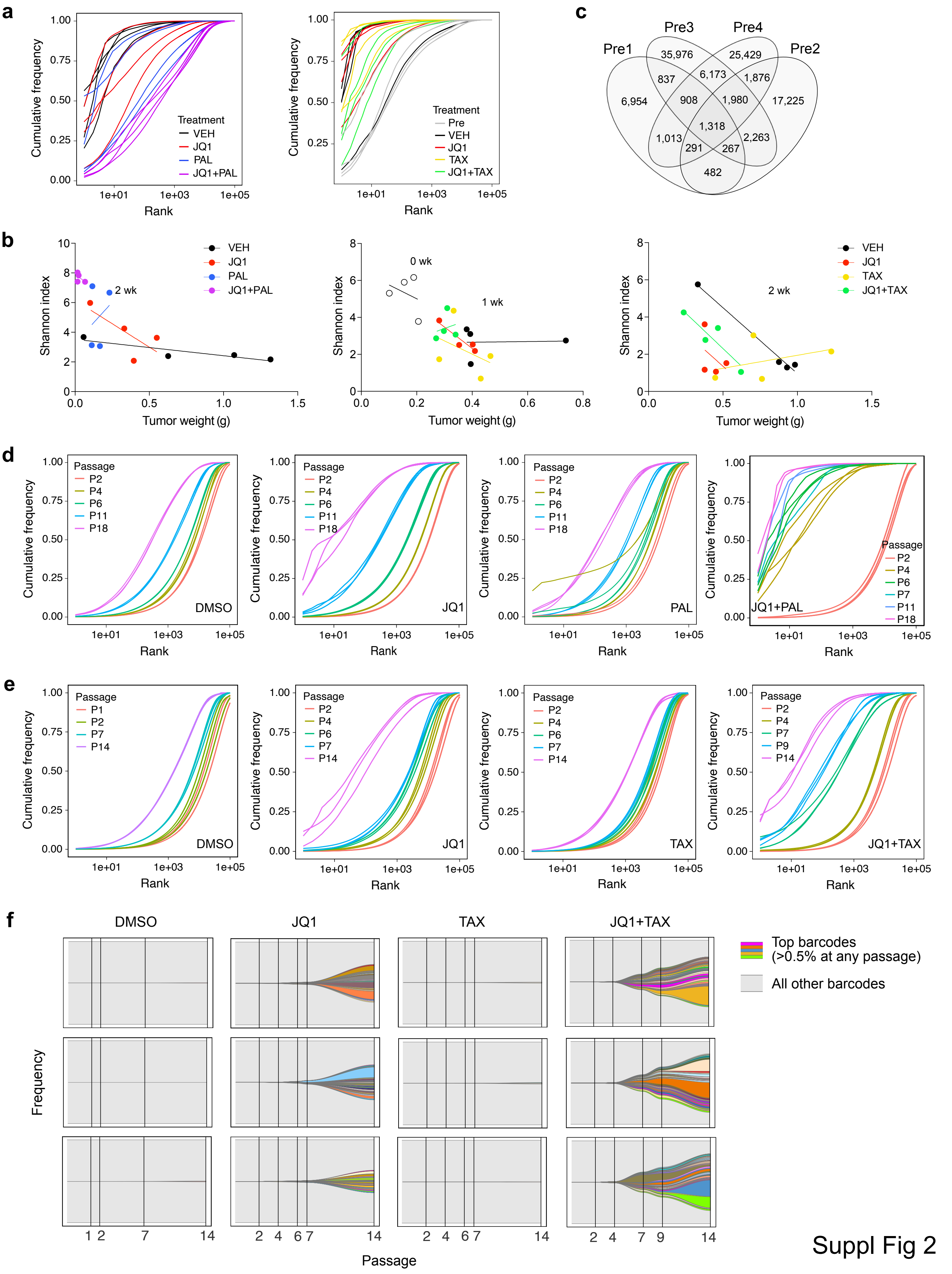

a
Cumulative frequency
Rank
Treatment
VEH
JQ1
PAL
JQ1+PAL
Pre
TAX
JQ1+TAX
b
Shannon index
Tumor weight (g)
2 wk
0 wk
1 wk
c
Pre1
Pre3
Pre4
Pre2
35,976
25,429
837
6,173
1,876
6,954
908
1,980
17,225
1,318
1,013
2,263
291
267
482
d
Passage
DMSO
JQ1
PAL
JQ1+PAL
e
TAX
JQ1+TAX
f
Top barcodes
(>0.5% at any passage)
All other barcodes
Frequency
Passage

Suppl Fig 2

Supplementary Figure 3. Proportions of shared barcodes between replicates selected with JQ1 combinations and simulated barcode diversities. **a**, Proportion of top barcodes (*x*-axis) in each replicate at the last passage that are unique to that replicate, shared between 2 replicates, or shared between all 3 replicates passaged in DMSO, JQ1, palbociclib (PAL), and JQ1+palbociclib. One JQ1+palbociclib treated replicate died out during the treatment course, so barcodes are only shared between 2 replicates. **b**, Proportion of top barcodes (*x*-axis) in each replicate at the last passage that are unique to that replicate, shared between 2 replicates, or shared between all 3 replicates passaged in DMSO, JQ1, paclitaxel (TAX), and JQ1+paclitaxel. **c**, Comparison between simulated data and experimental data of Shannon indices for cells passaged in JQ1, over a range of values for ρ and μ. Data are represented as mean ± SD, $n = 3$. **d**, Comparison between simulated data and experimental data of Shannon indices for cells passaged in palbociclib, over a range of values for ρ and μ. Data are represented as mean ± SD, $n = 3$.

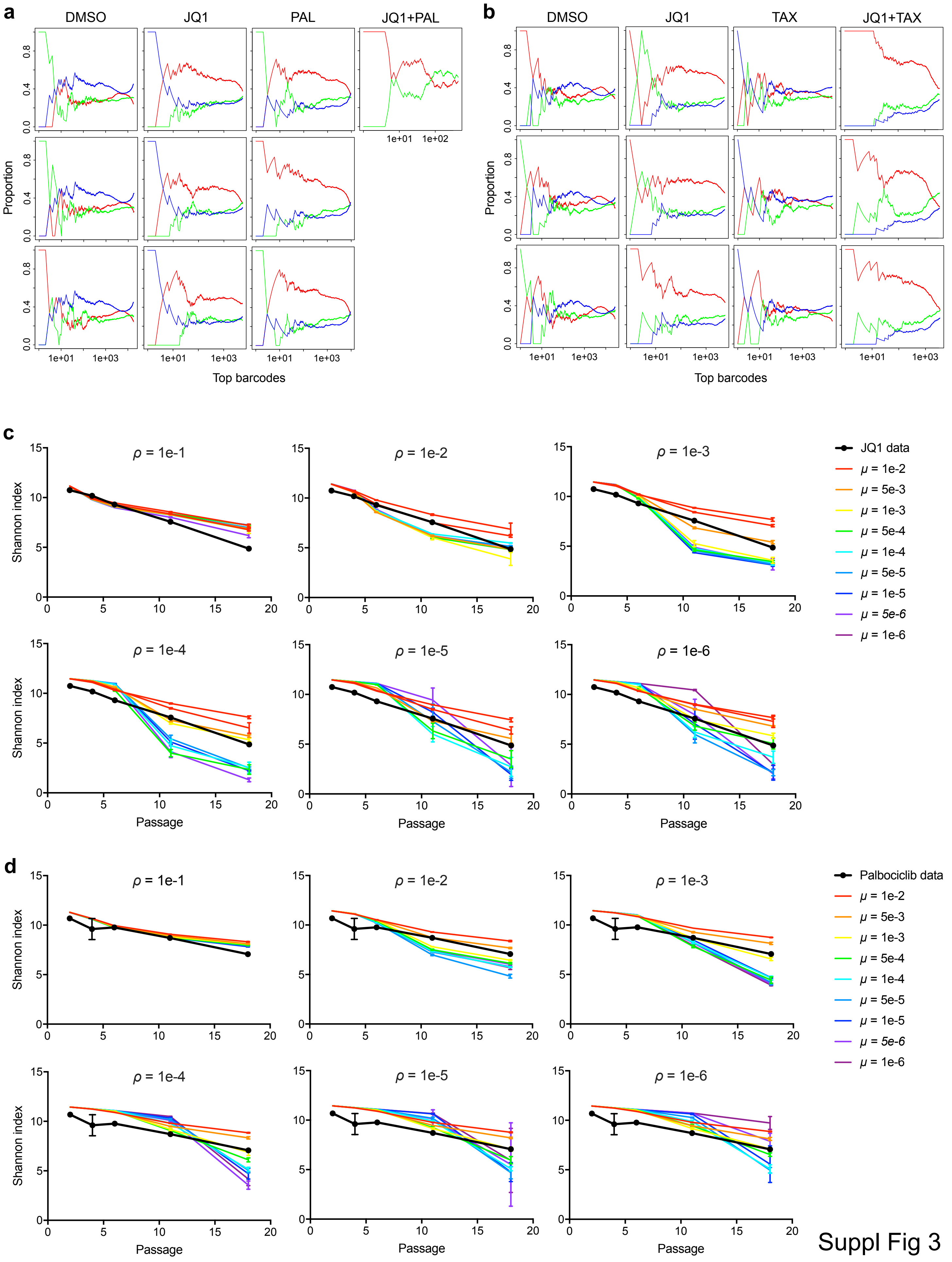

a
DMSO
JQ1
PAL
JQ1+PAL
Proportion
Top barcodes
b
DMSO
JQ1
TAX
JQ1+TAX
Proportion
Top barcodes
c
ρ = 1e-1
ρ = 1e-2
ρ = 1e-3
ρ = 1e-4
ρ = 1e-5
ρ = 1e-6
Shannon index
Passage
JQ1 data
μ = 1e-2
μ = 5e-3
μ = 1e-3
μ = 5e-4
μ = 1e-4
μ = 5e-5
μ = 1e-5
μ = 5e-6
μ = 1e-6
d
ρ = 1e-1
ρ = 1e-2
ρ = 1e-3
ρ = 1e-4
ρ = 1e-5
ρ = 1e-6
Shannon index
Passage
Palbociclib data
μ = 1e-2
μ = 5e-3
μ = 1e-3
μ = 5e-4
μ = 1e-4
μ = 5e-5
μ = 1e-5
μ = 5e-6
μ = 1e-6

Suppl Fig 3

Supplementary Figure 4. Simulated proportions of shared barcodes. a, Simulated proportions of top barcodes (*x*-axis) that are unique, shared between 2 replicates, or shared between all 3 replicates at the last passage of JQ1, over a range of values for ρ and μ. **b**, Simulated proportions of top barcodes (*x*-axis) that are unique, shared between 2 replicates, or shared between all 3 replicates at the last passage of palbociclib, over a range of values for ρ and μ.

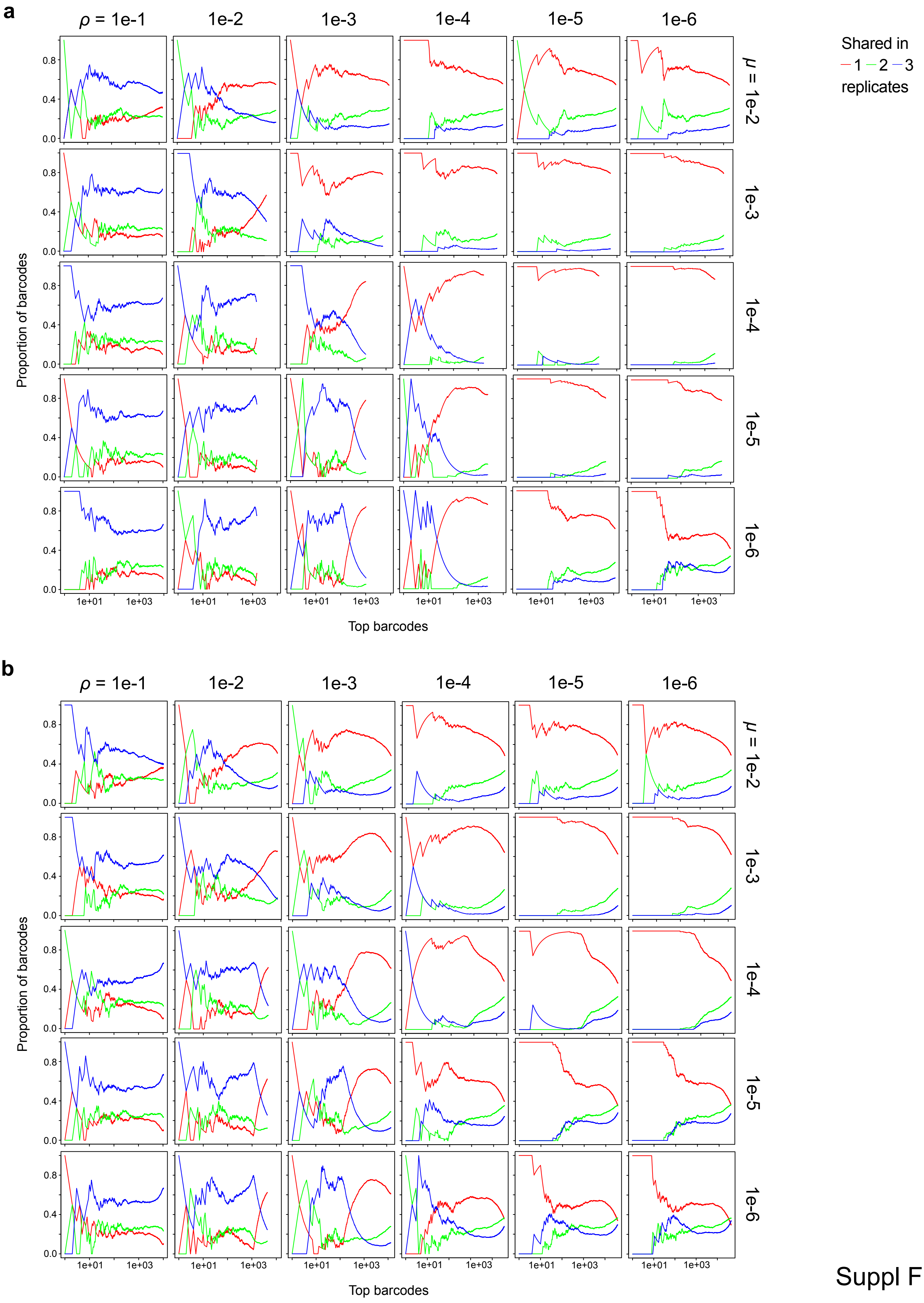
a
ρ = 1e-1
1e-2
1e-3
1e-4
1e-5
1e-6
μ = 1e-2
1e-3
1e-4
1e-5
1e-6
Shared in
1 2 3
replicates
Proportion of barcodes
0.0
0.4
0.8
1e+01
1e+03
Top barcodes
b
ρ = 1e-1
1e-2
1e-3
1e-4
1e-5
1e-6
μ = 1e-2
1e-3
1e-4
1e-5
1e-6
Proportion of barcodes
Top barcodes

Suppl Fig 4

Supplementary Figure 5. Comparison between simulated and experimental proportions of shared barcodes. a, Comparison between simulated data and experimental data of proportions of top barcodes (*x*-axis) that are unique, shared between 2 replicates, or shared between all 3 replicates at the last passage of JQ1, over a narrowed down range of values for ρ and μ. Simulated distributions are represented as mean ± SD, *n* = 5 independent simulations with 3 replicates each. **b**, Comparison between simulated data and experimental data of proportions of top barcodes (*x*-axis) that are unique, shared between 2 replicates, or shared between all 3 replicates at the last passage of palbociclib, over a narrowed down range of values for for ρ and μ. Simulated distributions are represented as mean ± SD, *n* = 5 independent simulations with 3 replicates each.

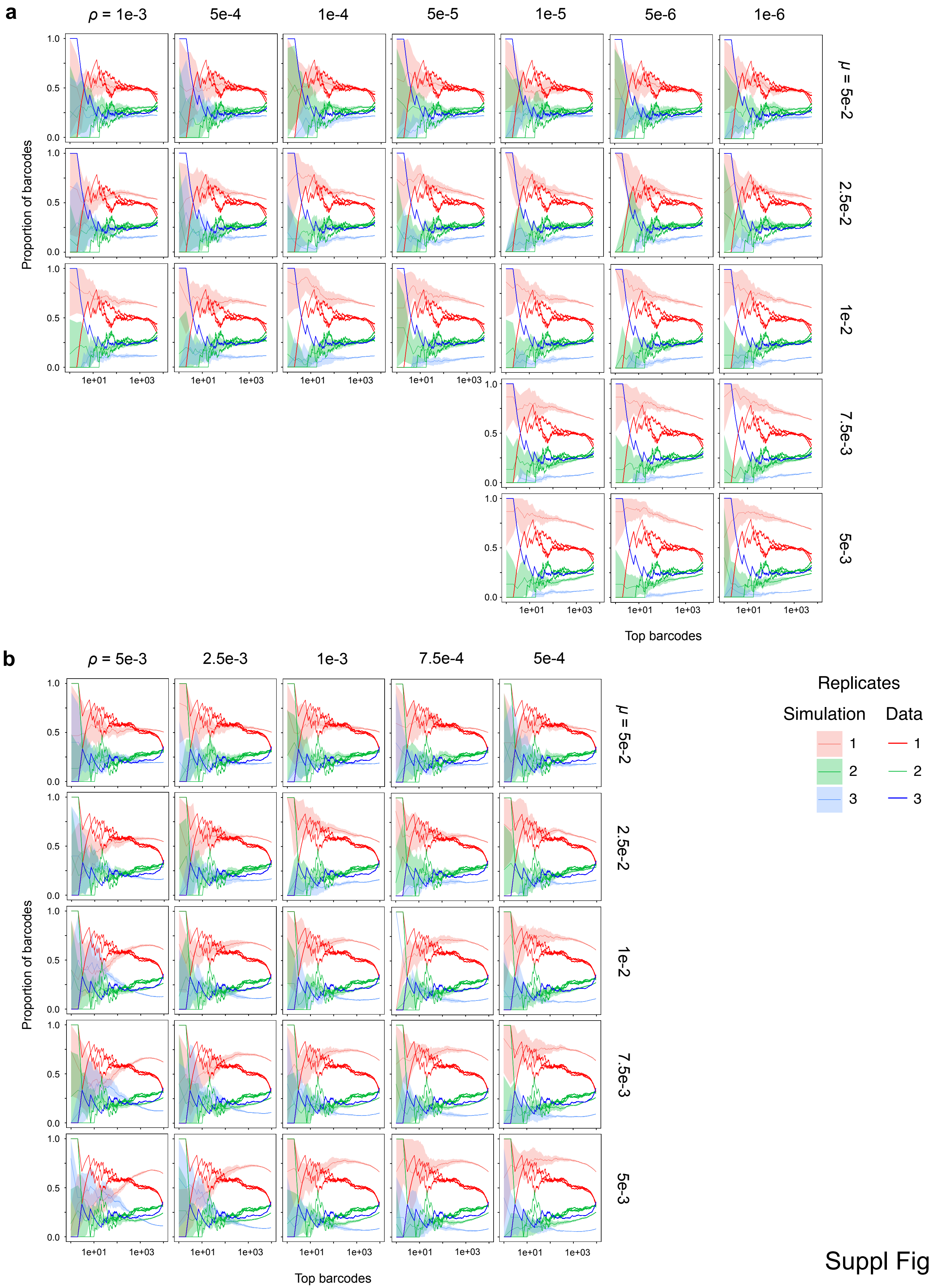
a
ρ = 1e-3
5e-4
1e-4
5e-5
1e-5
5e-6
1e-6
μ = 5e-2
2.5e-2
1e-2
7.5e-3
5e-3
Proportion of barcodes
Top barcodes
1.0
0.5
0.0
1e+01
1e+03
b
ρ = 5e-3
2.5e-3
1e-3
7.5e-4
5e-4
μ = 5e-2
2.5e-2
1e-2
7.5e-3
5e-3
Replicates
Simulation
Data
1
2
3

Suppl Fig 5

Supplementary Figure 6. JQ1 and palbociclib induce increased ploidy through cell division failure. a, Representative karyotypes of a minority of cells that was tetraploid after passaging in DMSO and of cells after passaging in JQ1 and palbociclib (PAL). Ranges of chromosome numbers and numbers of metaphase spreads counted are shown. Arrows indicate abnormal chromosomes. **b**, Gating strategy for flow cytometry analysis of DNA content in G1 and S/G2M fractions of Hoechst-stained FUCCI-labeled cells (shown are DMSO-treated cells). **c**, Histograms of DNA content by flow cytometry in Hoechst-stained FUCCI-labeled G1 SUM149, CAL-51, and MCF10A cells treated with DMSO, JQ1, palbociclib, and JQ1+palbociclib for 7 days. Dihydrocytochalasin B (DCB)-induced tetraploid cells were used as positive controls. Table indicates proportion of cells in the tetraploid gate. **d,** Histograms of DNA content by flow cytometry in a panel of TNBC cells treated with DMSO, JQ1, palbociclib, and JQ1+palbociclib for 7 days and stained with Hoechst. Dihydrocytochalasin B (DCB)-induced tetraploid cells were used as positive controls. Table indicates proportion of cells in the euploid gate, i.e. those with the baseline ploidy for each cell line. Note: these cell lines were not FUCCI-labeled; thus, the analysis includes cells at any cell cycle phase. **e**, TNBC subtype, *RB1* and p53 status, and average ploidy of cell lines tested in this study.

Suppl Fig 6

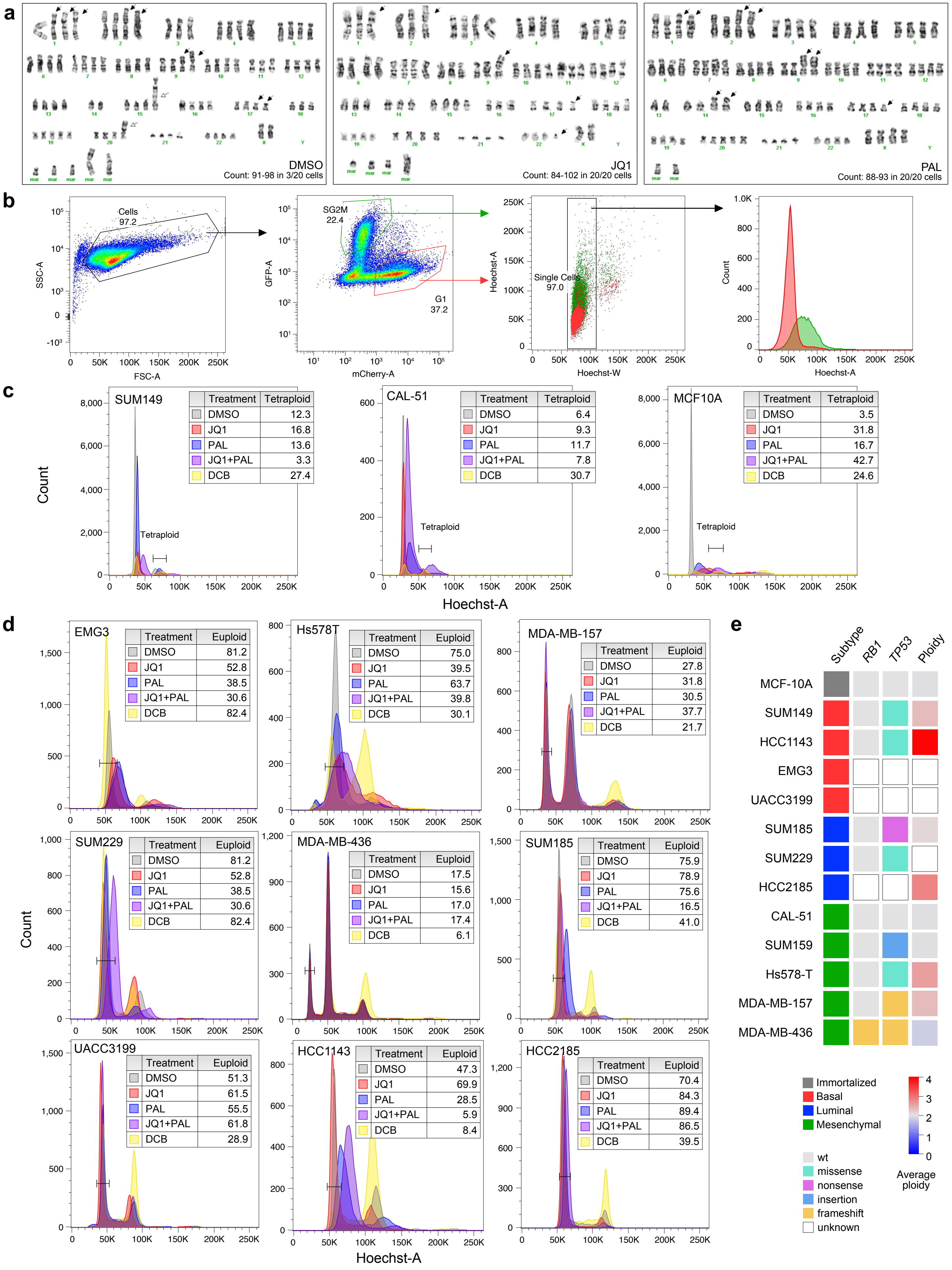
a
DMSO
Count: 91-98 in 3/20 cells
JQ1
Count: 84-102 in 20/20 cells
PAL
Count: 88-93 in 20/20 cells
b
Cells 97.2
SSC-A
FSC-A
SG2M 22.4
G1 37.2
GFP-A
mCherry-A
Single Cells 97.0
Hoechst-A
Hoechst-W
Count
Hoechst-A
c
SUM149
Treatment | Tetraploid
DMSO | 12.3
JQ1 | 16.8
PAL | 13.6
JQ1+PAL | 3.3
DCB | 27.4
Tetraploid
CAL-51
Treatment | Tetraploid
DMSO | 6.4
JQ1 | 9.3
PAL | 11.7
JQ1+PAL | 7.8
DCB | 30.7
Tetraploid
MCF10A
Treatment | Tetraploid
DMSO | 3.5
JQ1 | 31.8
PAL | 16.7
JQ1+PAL | 42.7
DCB | 24.6
Tetraploid
Count
Hoechst-A
d
EMG3
Treatment | Euploid
DMSO | 81.2
JQ1 | 52.8
PAL | 38.5
JQ1+PAL | 30.6
DCB | 82.4
Hs578T
Treatment | Euploid
DMSO | 75.0
JQ1 | 39.5
PAL | 63.7
JQ1+PAL | 39.8
DCB | 30.1
MDA-MB-157
Treatment | Euploid
DMSO | 27.8
JQ1 | 31.8
PAL | 30.5
JQ1+PAL | 37.7
DCB | 21.7
SUM229
Treatment | Euploid
DMSO | 81.2
JQ1 | 52.8
PAL | 38.5
JQ1+PAL | 30.6
DCB | 82.4
MDA-MB-436
Treatment | Euploid
DMSO | 17.5
JQ1 | 15.6
PAL | 17.0
JQ1+PAL | 17.4
DCB | 6.1
SUM185
Treatment | Euploid
DMSO | 75.9
JQ1 | 78.9
PAL | 75.6
JQ1+PAL | 16.5
DCB | 41.0
UACC3199
Treatment | Euploid
DMSO | 51.3
JQ1 | 61.5
PAL | 55.5
JQ1+PAL | 61.8
DCB | 28.9
HCC1143
Treatment | Euploid
DMSO | 47.3
JQ1 | 69.9
PAL | 28.5
JQ1+PAL | 5.9
DCB | 8.4
HCC2185
Treatment | Euploid
DMSO | 70.4
JQ1 | 84.3
PAL | 89.4
JQ1+PAL | 86.5
DCB | 39.5
Count
Hoechst-A
e
Subtype
RB1
TP53
Ploidy
MCF-10A
SUM149
HCC1143
EMG3
UACC3199
SUM185
SUM229
HCC2185
CAL-51
SUM159
Hs578-T
MDA-MB-157
MDA-MB-436
Immortalized
Basal
Luminal
Mesenchymal
wt
missense
nonsense
insertion
frameshift
unknown
Average ploidy

Supplementary Figure 7. JQ1 and CDK4/6 inhibitors induce increased ploidy through cell division failure. **a**, Metaphase spreads of SUM159 cells following treatment with DMSO, JQ1, palbociclib (PAL), and JQ1+palbociclib for 7 days. Counts indicate numbers of chromosomes. Images are representative of one experiment with 20 cells counted per group. **b**, Histograms of DNA content by flow cytometry in Hoechst-stained FUCCI-labeled G1 SUM159 cells treated with DMSO, JQ1, abemaciclib (AB), JQ1+abemaciclib, ribociclib (RIB), and JQ1+ribociclib for 7 days. Dihydrocytochalasin B (DCB)-induced tetraploid cells were used as positive controls. Table indicates proportion of cells in the tetraploid gate. **c,** Western blots show CDK4 and CDK6 levels in SUM159 and CAL-51 cells after CDK4, CDK6, and CDK4/6 siRNA knockdown. **d**, Histograms of DNA content by flow cytometry in 7-AAD-stained SUM159 and CAL-51 cells 6 days post-transfection with scramble, CDK4, CDK6, or CDK4 and 6 siRNA. Note: these cell lines were not FUCCI-labeled; thus, the analysis includes cells at any cell cycle phase. **e**, Image shows co-cultured RFP^+ and GFP^+ cells. Arrows indicate fused (GFP^+RFP^+, yellow) cells. Image is representative of two independent experiments. Scale bar represents 500 µm. **f**, Gating strategy for flow cytometry analysis of fused cells in co-cultures of GFP^+ and RFP^+ cells (shown are DMSO-treated cells). **g**, Scatterplots of flow cytometry analysis of co-cultured GFP^+ and RFP^+ cells quantifying the proportion of fused (GFP^+RFP^+) cells following 7 days of treatment. **h,** Dose response curves for JQ1 and palbociclib of diploid cells (parental and FUCCI) and sorted cells enriched for tetraploids (FUCCI-tet from FUCCI G1 tetraploids and GFP/RFP-tet from GFP^+RFP^+ fusions). Data are represented as mean ± SD, n = 4. Source data are provided as a Source Data file. **i**, Levels of synergism between JQ1 and palbociclib in diploid cells (parental and FUCCI) and sorted cells enriched for tetraploids (FUCCI-tet and GFP^+RFP^+-tet). Each point represents CI for one pair of concentrations, averaged over 8 replicates. CI = 1 additive, CI < 1 synergistic, and CI > 1 antagonistic. Source data are provided as a Source Data file. **j**, Histograms of DNA content by flow cytometry in Hoechst-stained SUM159 homofusions following treatment with DMSO, JQ1, palbociclib, and JQ1+palbociclib for 7 days. Table indicates proportion of cells in the octaploid gate. Note: these cell lines were not FUCCI-labeled; thus, the analysis includes cells at any cell cycle phase.

Suppl Fig 7

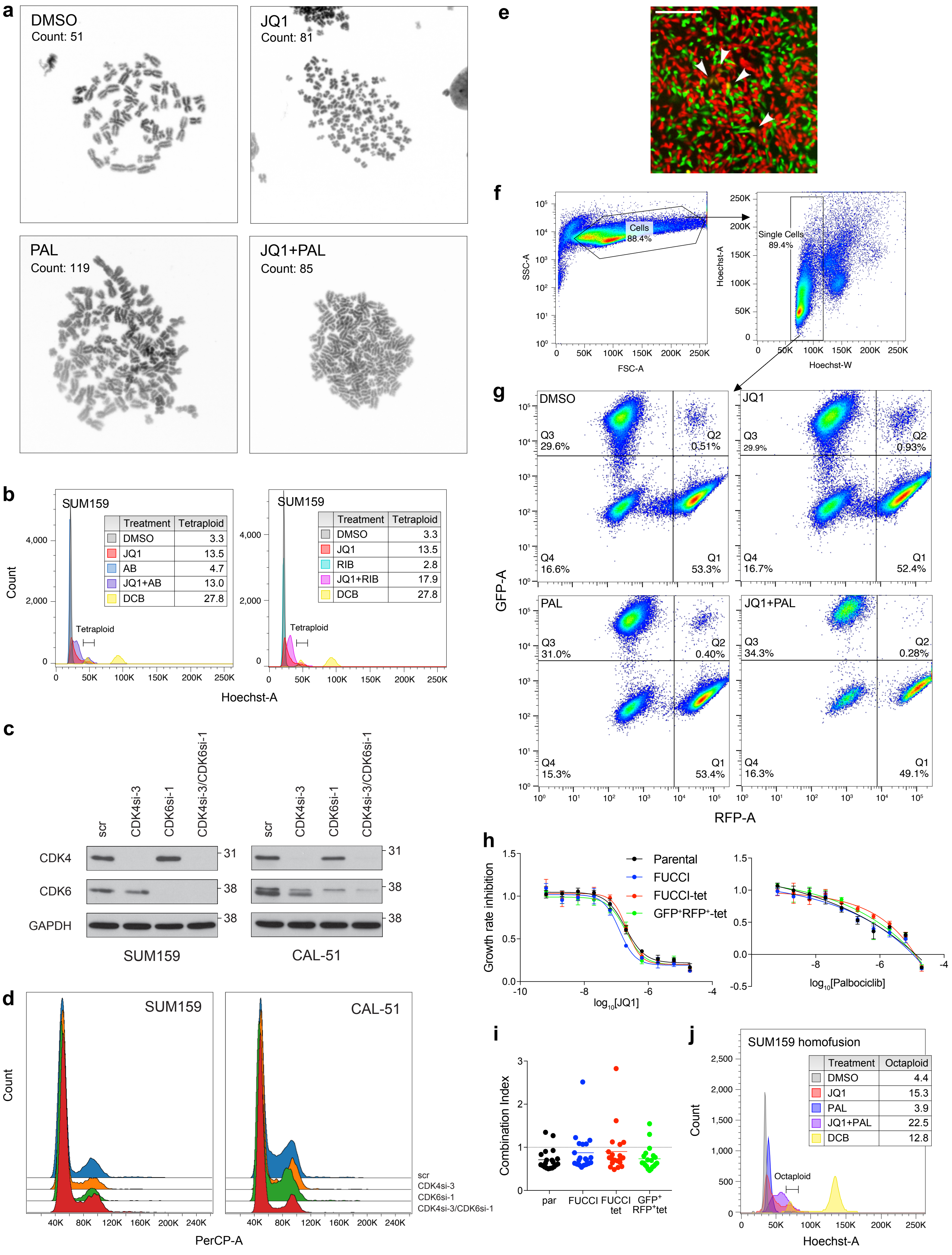
a
DMSO
Count: 51
JQ1
Count: 81
PAL
Count: 119
JQ1+PAL
Count: 85
b
SUM159
Treatment Tetraploid
DMSO 3.3
JQ1 13.5
AB 4.7
JQ1+AB 13.0
DCB 27.8
Tetraploid
SUM159
Treatment Tetraploid
DMSO 3.3
JQ1 13.5
RIB 2.8
JQ1+RIB 17.9
DCB 27.8
Tetraploid
Count
Hoechst-A
c
scr
CDK4si-3
CDK6si-1
CDK4si-3/CDK6si-1
CDK4
CDK6
GAPDH
31
38
38
SUM159
CAL-51
d
SUM159
CAL-51
Count
scr
CDK4si-3
CDK6si-1
CDK4si-3/CDK6si-1
PerCP-A
e
f
Cells
88.4%
SSC-A
FSC-A
Single Cells
89.4%
Hoechst-A
Hoechst-W
g
DMSO
Q3 29.6%
Q2 0.51%
Q4 16.6%
Q1 53.3%
JQ1
Q3 29.9%
Q2 0.93%
Q4 16.7%
Q1 52.4%
PAL
Q3 31.0%
Q2 0.40%
Q4 15.3%
Q1 53.4%
JQ1+PAL
Q3 34.3%
Q2 0.28%
Q4 16.3%
Q1 49.1%
GFP-A
RFP-A
h
Parental
FUCCI
FUCCI-tet
GFP⁺RFP⁺-tet
Growth rate inhibition
log10[JQ1]
log10[Palbociclib]
i
Combination Index
par
FUCCI
FUCCI tet
GFP⁺ RFP⁺tet
j
SUM159 homofusion
Treatment Octaploid
DMSO 4.4
JQ1 15.3
PAL 3.9
JQ1+PAL 22.5
DCB 12.8
Octaploid
Count
Hoechst-A

Supplementary Figure 8. RNA-seq and single cell RNA-seq analysis of post-selection cells. a, Heat map of differentially expressed genes in the core cell cycle process network by bulk RNA-seq of post JQ1, palbociclib (PAL), JQ1+palbociclib (JQ1+PAL), and JQ1+paclitaxel (JQ1+TAX) selected cells compared with post-DMSO selected cells, as well as tetraploid homofusion cells compared with diploid parental cells. **b**, Separate *t*-SNE plots of cells from the pre-treatment population (Pre) and from the post-selection populations of DMSO, JQ1, palbociclib (PAL), paclitaxel (TAX), and JQ1+paclitaxel (JQ1+TAX) by single-cell RNA-seq. Each point represents one single cell.

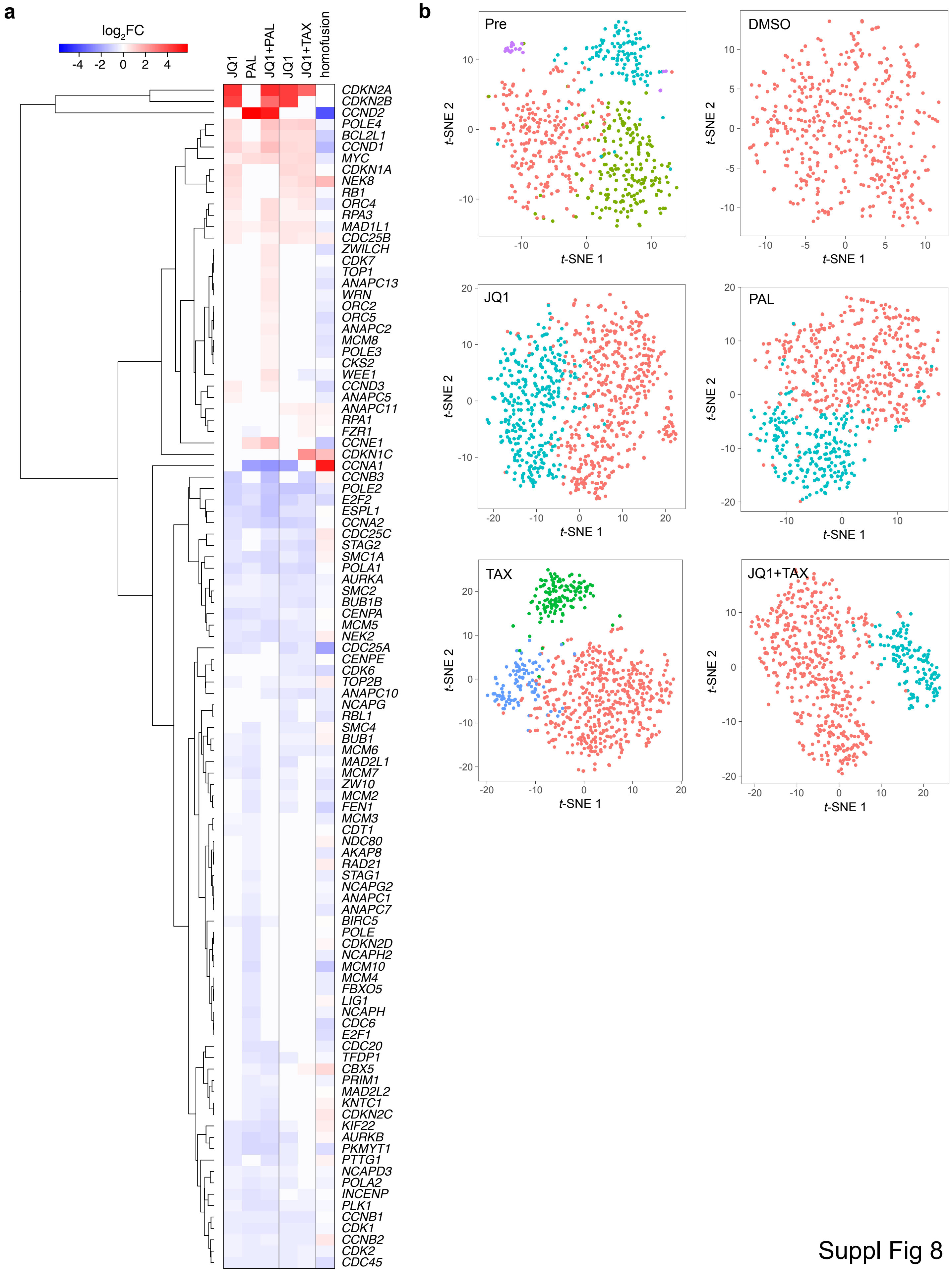

a
log$_2$FC
-4 -2 0 2 4
JQ1
PAL
JQ1+PAL
JQ1
JQ1+TAX
homofusion
CDKN2A
CDKN2B
CCND2
POLE4
BCL2L1
CCND1
MYC
CDKN1A
NEK8
RB1
ORC4
RPA3
MAD1L1
CDC25B
ZWILCH
CDK7
TOP1
ANAPC13
WRN
ORC2
ORC5
ANAPC2
MCM8
POLE3
CKS2
WEE1
CCND3
ANAPC5
ANAPC11
RPA1
FZR1
CCNE1
CDKN1C
CCNA1
CCNB3
POLE2
E2F2
ESPL1
CCNA2
CDC25C
STAG2
SMC1A
POLA1
AURKA
SMC2
BUB1B
CENPA
MCM5
NEK2
CDC25A
CENPE
CDK6
TOP2B
ANAPC10
NCAPG
RBL1
SMC4
BUB1
MCM6
MAD2L1
MCM7
ZW10
MCM2
FEN1
MCM3
CDT1
NDC80
AKAP8
RAD21
STAG1
NCAPG2
ANAPC1
ANAPC7
BIRC5
POLE
CDKN2D
NCAPH2
MCM10
MCM4
FBXO5
LIG1
NCAPH
CDC6
E2F1
CDC20
TFDP1
CBX5
PRIM1
MAD2L2
KNTC1
CDKN2C
KIF22
AURKB
PKMYT1
PTTG1
NCAPD3
POLA2
INCENP
PLK1
CCNB1
CDK1
CCNB2
CDK2
CDC45
b
Pre
DMSO
JQ1
PAL
TAX
JQ1+TAX
t-SNE 1
t-SNE 2

Suppl Fig 8
