# Supplementary material for: Acquired resistance to combined BET and CDK4/6 inhibition in triple-negative breast cancer
Source: Nat Commun. 2020 May 11;11:2350. doi: 10.1038/s41467-020-16170-3 (PMC7214447; doi:10.1038/s41467-020-16170-3)
Supplement: Supplementary file 2 — Description of Additional Supplementary Files [file 41467_2020_16170_MOESM2_ESM.docx]

Description of Additional Supplementary Files

**Supplementary Data 1.** Variants in whole exome sequencing. Single-nucleotide variants (SNVs), indels, and copy-number variants (CNVs) detected by whole exome sequencing of post-selection SUM159 cells. SNVs and indels are filtered for differences between DMSO-selected samples vs. pretreatment samples and for differences between JQ1, palbociclib, paclitaxel, JQ1+palbociclib, and JQ1+paclitaxel-selected samples vs. DMSO-selected samples.

**Supplementary Data S2.** RNA-seq differentially expressed genes. Differentially expressed genes by RNA-seq between JQ1, palbociclib, and JQ1+palbociclib-selected cells vs. DMSO-selected cells.

**Supplementary Data S3.** Single cell RNA-seq differentially expressed genes in JQ1+palbociclib-selected cells. Differentially expressed genes in the four clusters of JQ1+palbociclib-selected cells by single cell RNA-seq.

**Supplementary Video 1.** Normal mitosis. Representative video of DMSO-treated SUM159 cell undergoing normal mitosis, as seen in Figure 5e. Cells were labeled with H2B-GFP and membraneTdTomato. Images were acquired at 10-minute intervals using a 20X objective. Time shown in hh:min after treatment.

**Supplementary Video 2.** Chromosomal missegregation during mitosis. Representative video of SUM159 cell treated with JQ1 undergoing chromosomal missegregation during mitosis to form multinucleated cells, as seen in Figure 5e. Imaging was performed as in Supplementary Video 1.

**Supplementary Video 3.** Chromosomal missegregation during mitosis. Representative video of SUM159 cell treated with palbociclib undergoing chromosomal missegregation during mitosis to form multinucleated cells, as seen in Figure 5e. Imaging was performed as in Supplementary Video 1.

**Supplementary Video 4.** Mitotic delay and cytokinesis failure. Representative video of SUM159 cell experiencing mitotic delay and cytokinesis failure (arrow), as in after treatment with JQ1 or palbociclib (JQ1-treated cell is shown). Imaging was performed as in Supplementary Video 1.

**Supplementary Video 5.** Block in mitotic progression and multinuclei formation. Representative video of JQ1+palbociclib-treated cell experiencing block in mitotic progression and formation of multinuclei (arrow), as seen in Figure 5e. Imaging was performed as in Supplementary Video 1.

**Supplementary Video 6.** Block in mitotic progression and karyokinesis failure. Representative video of JQ1+palbociclib-treated cell experiencing block in mitotic progression and karyokinesis failure (arrow). Imaging was performed as in Supplementary Video 1.
