# Supplementary material for: Acquired resistance to combined BET and CDK4/6 inhibition in triple-negative breast cancer
Source: Nat Commun. 2020 May 11;11:2350. doi: 10.1038/s41467-020-16170-3 (PMC7214447; doi:10.1038/s41467-020-16170-3)
Supplement: Supplementary file 12 — Reporting Summary [file 41467_2020_16170_MOESM12_ESM.pdf]

nature research

Corresponding author(s): Kornelia Polyak, Franziska Michor

Last updated by author(s): Apr 10, 2020

Reporting Summary

Nature Research wishes to improve the reproducibility of the work that we publish. This form provides structure for consistency and transparency in reporting. For further information on Nature Research policies, seeAuthors & Referees and theEditorial Policy Checklist .

Statistics

For all statistical analyses, confirm that the following items are present in the figure legend, table legend, main text, or Methods section.

n/a	Confirmed	
☐	☒	The exact sample size (*n*) for each experimental group/condition, given as a discrete number and unit of measurement
☐	☒	A statement on whether measurements were taken from distinct samples or whether the same sample was measured repeatedly
☐	☒	The statistical test(s) used AND whether they are one- or two-sided<br>*Only common tests should be described solely by name; describe more complex techniques in the Methods section.*
☐	☒	A description of all covariates tested
☐	☒	A description of any assumptions or corrections, such as tests of normality and adjustment for multiple comparisons
☐	☒	A full description of the statistical parameters including central tendency (e.g. means) or other basic estimates (e.g. regression coefficient) AND variation (e.g. standard deviation) or associated estimates of uncertainty (e.g. confidence intervals)
☐	☒	For null hypothesis testing, the test statistic (e.g. *F*, *t*, *r*) with confidence intervals, effect sizes, degrees of freedom and *P* value noted<br>*Give P values as exact values whenever suitable.*
☒	☐	For Bayesian analysis, information on the choice of priors and Markov chain Monte Carlo settings
☒	☐	For hierarchical and complex designs, identification of the appropriate level for tests and full reporting of outcomes
☐	☒	Estimates of effect sizes (e.g. Cohen's *d*, Pearson's *r*), indicating how they were calculated

Our web collection on statistics for biologists contains articles on many of the points above.

Software and code

Policy information about availability of computer code

Data collection	Cell counts were collected using the Celigo Imaging Cytometer (Nexcelom). Images were acquired by confocal microscopy (Leica SP5X laser scanning confocal microscope for immunofluorescence or TE2000-E2 inverted microscope with MetaMorph software (v7.10.2.240) for live imaging). Flow cytometry data were acquired on an LSRFortessa cytometer (BD Biosciences). ddPCR data were collected using the QX200 Droplet Reader (Bio-Rad).
Data analysis	Statistical analyses were performed using GraphPad Prism (v8.1.1) or R (v3.6.2). Flow cytometry data were analyzed using FlowJo (v10) or Cytobank (v7.2.0). ddPCR data were analyzed using QuantaSoft (v1.7.4). RNAseq experiments were analyzed using STAR (v2.6.0a), HTSeq-count (v0.6.1p1), DESeq2 (v1.20.0), and MetaCore (v19.4). Single cell RNA-seq data were analyzed using CellRanger (v2.1.0) and Seurat (v2.3.4). Exome data were analyzed using Picard (v2.18.0), MuTect (v1.1.4), Variant Effect Predictor (v79), GATK (v1.65), OncoAnnotate (v1.3.0), and RobustCNV (in development).

For manuscripts utilizing custom algorithms or software that are central to the research but not yet described in published literature, software must be made available to editors/reviewers. We strongly encourage code deposition in a community repository (e.g. GitHub). See the Nature Research guidelines for submitting code & software for further information.

Data

Policy information about availability of data

All manuscripts must include a data availability statement. This statement should provide the following information, where applicable:

- Accession codes, unique identifiers, or web links for publicly available datasets
- A list of figures that have associated raw data
- A description of any restrictions on data availability

The barcode, exome, RNA-seq, and single cell RNA-seq data have been deposited in GEO under ID code GSE131986 (https://www.ncbi.nlm.nih.gov/geo/query/acc.cgi?acc=GSE131986). The source data underlying Figs. 1a-b, f, 3b, 4, 7, and Supplementary Fig. 7h-i are provided as a Source Data file.

Field-specific reporting

Please select the one below that is the best fit for your research. If you are not sure, read the appropriate sections before making your selection.

☒ Life sciences ☐ Behavioural & social sciences ☐ Ecological, evolutionary & environmental sciences

For a reference copy of the document with all sections, see nature.com/documents/nr-reporting-summary-flat.pdf

Life sciences study design

All studies must disclose on these points even when the disclosure is negative.

Sample size	Sample sizes were chosen based on our prior studies using the same types of assays, as well as published literature, to ensure statistically significant results. Proliferation and barcode studies were performed in triplicates (Hinohara et al. Cancer Cell 2018). Synergy studies were performed with 8 replicates (Shu et al. Nature 2016). In vivo studies were performed with 5 mice per treatment group with two tumors each, which we estimated would account for variability in tumor growth (Marusyk et al. Nature 2014 and Hu et al. Cancer Cell 2008). RNA-seq was performed on duplicates and exome sequencing on pooled triplicates (Shu et al. Nature 2016 and Hinohara et al. Cancer Cell 2018). Single-cell RNA-seq was performed on 600-1000 cells pooled from triplicates per treatment group (Janiszewska et al. NCB 2019 and Shu et al. Molecular Cell 2020).
Data exclusions	No data have been excluded.
Replication	Proliferation, synergy, flow cytometry, and live imaging assays were reproduced in three independent experiments. Simulations were performed on 5 independent runs. All attempts at replication were successful. Barcode selection, animal studies, and sequencing of these samples were performed once due to time and cost limitations.
Randomization	Mice were randomized to treatment groups after they developed palpable tumors. Randomization was not relevant for other experiments, as they were performed in cell lines.
Blinding	Mouse experiments were repeated by technical assistants in the Lurie Family Imaging Center who were blinded to the identity of the samples. Similarly, some RNA-seq and exome sequencing analysis was performed by bioinformaticians blinded to the identity of the samples. For proliferation, synergy, barcode, and flow cytometry studies, blinding was not possible, since samples had to be labeled. However, these assays were performed by multiple independent personnel participating in the study at different times during the project with the same results.

Reporting for specific materials, systems and methods

We require information from authors about some types of materials, experimental systems and methods used in many studies. Here, indicate whether each material, system or method listed is relevant to your study. If you are not sure if a list item applies to your research, read the appropriate section before selecting a response.

Materials & experimental systems

n/a	Involved in the study	
☐	☒	Antibodies
☐	☒	Eukaryotic cell lines
☒	☐	Palaeontology
☐	☒	Animals and other organisms
☒	☐	Human research participants
☒	☐	Clinical data

Methods

n/a	Involved in the study	
☒	☐	ChIP-seq
☐	☒	Flow cytometry
☒	☐	MRI-based neuroimaging

Antibodies

Antibodies used	recombinant anti-cyclin D1 (Abcam, ab134175, EPR2241); anti-cleaved caspase-3 Asp175 (Cell Signaling Technology, 9661); anti-histone H3 phospho S10 (Abcam, ab5176); anti-α-tubulin (Sigma-Aldrich, T9026, DM1A); anti-phospho-Rb S780 (Cell Signaling Technology, 8180, D59B7); anti-CDK4 (Thermo Fisher Scientific, MS616P1, DCS-31 and DCS-35); anti-CDK6 (Abcam, ab54576); anti-β-actin (Sigma-Aldrich, A2228, AC-74); anti-GAPDH (Cell Signaling Technology, 5174, D16H11); anti-goat anti-rabbit IgG highly cross-adsorbed secondary antibody, Alexa Fluor 647 (Thermo Fisher Scientific, A21245); goat anti-rabbit IgG highly cross-adsorbed secondary antibody, Alexa Fluor 488 (Thermo Fisher Scientific, A11008); goat anti-mouse IgG1 cross-adsorbed secondary antibody, Alexa Fluor 594 (Thermo Fisher Scientific, A21125); Zenon Alexa Fluor 488 rabbit IgG labeling kit (Thermo Fisher Scientific, Z25302); Zenon Alexa Fluor 555 rabbit IgG labeling kit (Thermo Fisher Scientific, Z25305); goat anti-mouse IgG, HRP (Thermo Fisher Scientific, 32430); goat anti-rabbit, HRP (Fisher Scientific, 656120)
Validation	Anti-cyclin D1, cleaved caspase-3, pHistone H3, and α-tubulin antibodies were validated on cell-line derived xenografts with known expression of the marker. Anti-pRB, CDK4, and CDK6 antibodies were validated using CRISPR KO cell lines.

Eukaryotic cell lines

Policy information about cell lines

Cell line source(s)	SUM159, SUM149, SUM229, and SUM185 breast cancer cells were obtained from Steve Ethier (University of Michigan). CAL-51 cells were obtained from DSMZ. MCF10A, Hs578T, MDA-MB-157, MDA-MB-436, and HCC1143 cells were obtained from ATCC. EMG3 cells were obtained from Eva Matouskova (Academy of Sciences of the Czech Republic). UACC3199 cells were obtained from University of Arizona and are available from ATCC. HCC2185 cells were obtained from Adi Gazdar (UT Southwestern).
Authentication	The identity of the cell lines was confirmed based on STR and exome-seq analyses.
Mycoplasma contamination	Cell lines were routinely tested for mycoplasma and rodent pathogen contamination. No contamination was found at any time point.
Commonly misidentified lines (See ICLAC register)	No commonly misidentified lines were used in this study.

Animals and other organisms

Policy information about studies involving animals; ARRIVE guidelines recommended for reporting animal research

Laboratory animals	In vivo studies were conducted using 6-week-old female immunodeficient NOD.Cg-PrkdcscidIl2rgtm1Sug/JicTac (NOG) mice (Taconic) or NOD.Cg-PrkdcscidIl2rgtmWjl/SzJ (NSG) mice (Jackson Laboratory). Mice were housed 5 to a cage with ad libitum access to food and water in 20˚C ambient temperature, 40-50% humidity, and 12-hour light/12-hour dark cycle.
Wild animals	No wild animals were used in this study.
Field-collected samples	No field-collected samples were used in this study.
Ethics oversight	Animal studies were performed according to protocol 11-023 or by the Lurie Family Imaging Center according to protocol 04-111, approved by the Dana-Farber Cancer Institute Animal Care and Use Committee.

Note that full information on the approval of the study protocol must also be provided in the manuscript.

Flow Cytometry

Plots

Confirm that:

- [x] The axis labels state the marker and fluorochrome used (e.g. CD4-FITC).
- [x] The axis scales are clearly visible. Include numbers along axes only for bottom left plot of group (a 'group' is an analysis of identical markers).
- [x] All plots are contour plots with outliers or pseudocolor plots.
- [x] A numerical value for number of cells or percentage (with statistics) is provided.

Methodology

Sample preparation	For cell cycle analysis, cells were fixed overnight in 70% ethanol and then stained in 20 µg/mL propidium iodide (Thermo Fisher Scientific) with 0.2 mg/mL PureLink RNase A (Thermo Fisher Scientific) in 0.1% Triton X-100 (Sigma-Aldrich) for 30 minutes. For analysis of apoptosis, cells were stained using the Alexa Fluor 488 Annexin V/Dead Cell Apoptosis Kit as directed (Thermo Fisher Scientific). For ploidy studies, cells were stained with PBS with 10 µg/mL Hoechst 33342 (Sigma-Aldrich), 0.5% NP-40, and 3.7% paraformaldehyde overnight at 4˚C or with 7-AAD according to manufacturer’s instructions.
Instrument	Fluorescence intensities were acquired on an LSRFortessa cytometer (BD Biosciences).
Software	Data were analyzed using FlowJo.
Cell population abundance	We collected at least 10,000 cells.
Gating strategy	Cells with low FSC and SSC were gated out as dead cells and debris. For cell cycle analysis, doublets were gated out using high PI-A and PI-W, and cell cycle fractions were then gated using the Watson cell cycle model. For apoptosis analysis, gates were determined using the 4 populations seen in annexin V/PI stained cells treated with DMSO. FUCCI cells were gated by mCherry and GFP to identify G1 and SG2M cells, respectively, which were then gated by Hoechst-W to identify single cells. G1 tetraploid cells were then gated by Hoechst-A levels equivalent to that of DCB-treated controls. RFP/GFP co-cultured cells were gated by Hoechst-W to identify single cells and then by GFP and RFP for double-positive fusion cells. All gating strategies are shown in Supplementary Figures.

- [x] Tick this box to confirm that a figure exemplifying the gating strategy is provided in the Supplementary Information.
